# Integration of spatio-temporal variations of surface metabolomes and epibacterial communities highlights the importance of copper stress as a major factor shaping host-microbiota interactions within a Mediterranean seaweed holobiont

**DOI:** 10.1186/s40168-021-01124-8

**Published:** 2021-10-12

**Authors:** Benoît Paix, Nicolas Layglon, Christophe Le Poupon, Sébastien D’Onofrio, Benjamin Misson, Cédric Garnier, Gérald Culioli, Jean-François Briand

**Affiliations:** 1 Université de Toulon, Laboratoire MAPIEM, EA 4323 Toulon, France; 2 Present adress: Marine Biodiversity, Naturalis Biodiversity Center, Leiden, The Netherlands; 3 Université de Toulon, Aix Marseille Université, CNRS, IRD, Mediterranean Institute of Oceanography (MIO), UM 110 Toulon, France; 4 Present address: Institut Méditerranéen de Biodiversité et d’Ecologie marine et continentale (IMBE), UMR CNRS-IRD-Avignon Université-Aix-Marseille Université, Avignon, France

**Keywords:** Holobiont, Surface metabolome, Surface microbiota, Copper-stress, Temperature, Chemical interactions, Metabarcoding, Metabolomics

## Abstract

**Background:**

Although considered as holobionts, macroalgae and their surface microbiota share intimate interactions that are still poorly understood. Little is known on the effect of environmental parameters on the close relationships between the host and its surface-associated microbiota, and even more in a context of coastal pollutions. Therefore, the main objective of this study was to decipher the impact of local environmental parameters, especially trace metal concentrations, on an algal holobiont dynamics using the Phaeophyta *Taonia atomaria* as a model. Through a multidisciplinary multi-omics approach combining metabarcoding and untargeted LC-MS-based metabolomics, the epibacterial communities and the surface metabolome of *T. atomaria* were monitored along a spatio-temporal gradient in the bay of Toulon (Northwestern Mediterranean coast) and its surrounding. Indeed, this geographical area displays a well-described trace metal gradient particularly relevant to investigate the effect of such pollutants on marine organisms.

**Results:**

Epibacterial communities of *T. atomaria* exhibited a high specificity whatever the five environmentally contrasted collecting sites investigated on the NW Mediterranean coast. By integrating metabarcoding and metabolomics analyses, the holobiont dynamics varied as a whole. During the occurrence period of *T. atomaria*, epibacterial densities and *α*-diversity increased while the relative proportion of core communities decreased. Pioneer bacterial colonizers constituted a large part of the specific and core taxa, and their decrease might be linked to biofilm maturation through time. Then, the temporal increase of the *Roseobacter* was proposed to result from the higher temperature conditions, but also the increased production of dimethylsulfoniopropionate (DMSP) at the algal surface which could constitute of the source of carbon and sulfur for the catabolism pathways of these taxa. Finally, as a major result of this study, copper concentration constituted a key factor shaping the holobiont system. Thus, the higher expression of carotenoids suggested an oxidative stress which might result from an adaptation of the algal surface metabolome to high copper levels. In turn, this change in the surface metabolome composition could result in the selection of particular epibacterial taxa.

**Conclusion:**

We showed that associated epibacterial communities were highly specific to the algal host and that the holobiont dynamics varied as a whole. While temperature increase was confirmed to be one of the main parameters associated to *Taonia* dynamics, the originality of this study was highlighting copper-stress as a major driver of seaweed-epibacterial interactions. In a context of global change, this study brought new insights on the dynamics of a Mediterranean algal holobiont submitted to heavy anthropic pressures.

Video abstract

**Supplementary Information:**

The online version contains supplementary material available at 10.1186/s40168-021-01124-8.

## Introduction

In the marine environment, seaweed surfaces constitute niches for a large diversity of epiphytic organisms among which bacteria represent the main biological compartment [1–3]. Macroalgal epibacterial communities show a high specificity to their host [4–7], and chemical mediation appears to be involved in their relationships at the algal surface [8, 9]. Positive interactions have been demonstrated for the Chlorophyta *Ulva mutabilis* which releases a chemo-attracting signal (dimethylsulfoniopropionate, DMSP) for the *Roseovarius* sp. MS2 bacterial strain, which then uses the algal glycerol boundary layer as a carbon source and promotes the morphogenesis of the algal host [9, 10]. Negative interactions, like chemical defenses against epibacterial pathogens, were also observed [11, 12]. These results support that the algal host, mainly through the chemical production at its surface, could be strongly involved in the selection of a specific epiphytic microbiota that plays in turn major roles in the fitness of the alga. Considering intimate interactions between both, macroalgae and their surface microbiota may thus be considered together as a holobiont system [2, 13, 14].

However, little is known about the effect of environmental parameters on the relationships within the algal holobiont [15–17]. The production of halogenated furanones in *D. pulchra* decreases when the seawater temperature increases [18, 19], and this finding is linked to the occurrence of two bacterial pathogenic strains causing thalli bleaching [20–22] and subsequently an increased colonization of the algal surface [23]. For the Phaeophyceae *Fucus vesiculosus*, light and temperature are the main factors explaining a global temporal shift of the surface metabolome [24–26]. Moreover, a shift of its epiphytic bacterial community structure has been observed through experimental approaches using different temperature or salinity conditions [16, 27]. Overall, concerns have been raised for seaweed fitness due to the modification of their microbiota structure that can lead to dysbiosis under the effect of anticipated climate conditions, especially ocean temperature and acidification increases [28–34]. Global change, together with the increase of other anthropic pressures such as plastic pollution, trace metal contaminations, or eutrophication [35, 36], particularly threatens the Mediterranean Sea which is considered a hot-spot of marine biodiversity [35].

In this context, the algal holobiont studied here was the Phaeophyceae *Taonia atomaria* (Woodward) J. Agardh which is widely distributed on the North Western Atlantic and Mediterranean coasts. Some metabolites have been previously characterized at its surface and showed anti-adhesion properties against marine bacteria, supporting the hypothesis of a chemical selection of the epibacterial community [37, 38]. This consideration has been strengthened by the strong temporal co-variations observed between the epibacterial community and the surface metabolome of *T. atomaria* at a single site along the NW Mediterranean coast [14]. Nevertheless, investigation of environmental drivers that could control the holobiont dynamics appears necessary, as it has never been done before for *T. atomaria*, and more generally, rarely investigated in Mediterranean ecosystems.

In this study, we hypothesized that the selection of specific epibacterial communities could be the result of both the effect of the chemical production at the algal surface and environmental factors*.* Thus, a spatio-temporal multi-omics monitoring of the epiphytic microbiota and the algal surface metabolome, associated to the analysis of environmental parameters, was achieved during the annual occurrence of *T. atomaria* on the French NW Mediterranean coast (from February to March). Among environmental parameters, a special interest was focused on the effect of metal pollutants, such as copper, lead, or zinc. In this context, the bay of Toulon and its surroundings constitutes a privileged study site, as contrasted degrees of such pollution occur across space. On the one hand, the bay of Toulon is a semi-enclosed marine area characterized by historical and long-term gradients of trace metal contaminations, from military and civil harbors to the open sea [39–42]. More specifically, the occurrence of copper is mainly associated with antifouling coatings whereas lead contamination particularly arises from scuttled navy ships (World War II). On the other hand, the preserved marine area of the Port-Cros marine National Park is a close coastal environment showing very low levels of metal contaminations. Since metal pollution constitutes the predominant parameter changing across a close space-scale within this environment, studying site differences in this context will make possible to investigate the effect of this anthropic factor on the host-microbiome interactions of the algal communities living there.

## Materials and methods

### Sampling strategy and biological material

**Fig. 1:**
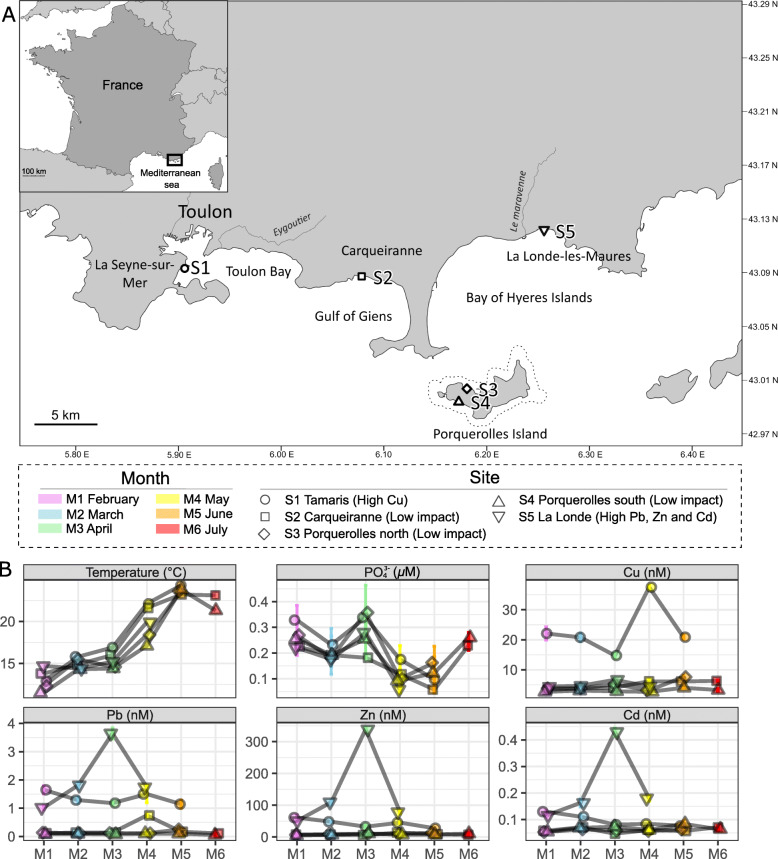
Sampling sites and main environmental parameters associated to the field survey. **A** Map with the location of the sampling sites. Dashed lines represented the limits of the preserved zone of the Porquerolles island within the Port-Cros marine National Park. **B** Plots associated to temporal variations of temperature, concentrations of PO_4_^3-^, and concentrations of dissolved trace metals (copper, lead, zinc, cadmium) measured from the seawater at each site

Sampling was performed monthly (~1m depth) on rocky substrates during the occurrence period of *T. atomaria* in 2017 (February to July) at five sites named S1 to S5 (Fig. 1) on the French Mediterranean coast. These sites were chosen considering the occurrence of *T. atomaria* and for their contrasted levels of trace metal contaminations, but also wind and currents (Fig. 1). S1 was located inside the bay of Toulon which is a highly anthropized area due to intensive military and commercial harbor activities [43]. S3 and S4 were located at Porquerolles Island in the marine preserved area of the Port-Cros National Park. These two sites differ in terms of wind exposure and currents, with S3 facing north in front of the Gulf of Giens coasts, while S4 is facing south towards the open sea (Fig. 1). S2 (a previously studied site [14, 37, 38, 44]) and S5 were chosen to represent shores in the Gulf of Giens and the Bay of Hyères Islands, respectively. The latter is closed to where a coastal river flows into the bay, but with an erratic functioning associated to massive rainfall. In addition, S3 and S5 are characterized by sandy bottoms while rocky shores are predominant at the other sampling sites (S1, S2, and S4). For each sampling, three thalli of *T. atomaria* were collected as replicates for metabolomics, flow cytometry, and metabarcoding, together with one neighboring rock and 5L of surrounding seawater for metabarcoding. Temperature, pH, salinity, and oxygen were measured using a multiparameter probe (Hydrolab® DS5X, Hatch Hydromet, USA) and surrounding seawater was also sampled for nutrients, total nitrogen (TN), dissolved organic carbon (DOC), and trace metal analyses [details in Supplementary Information (SI)].

Thalli were transported to the laboratory as described in [14]. For each triplicate, one frond was used for flow cytometry, a second for DNA extraction and sequencing, and a third for metabolomics.

### Chemical analysis of seawater

Nutrients (NO_3_^-^, PO_4_^3-^ and Si[OH]_4_) were analyzed using standard colorimetric methods and DOC and TN with a TOC-VCSH analyzer (Shimadzu, Noisiel, France) [43]. Dissolved and total concentrations of trace metal (lead, zinc, cadmium, and copper) were obtained by differential pulse anodic stripping voltammetry (DPASV) (details in SI).

### Flow cytometry analyses

Epibiotic cells were collected at the surface of algal samples by gently scraping the thalli with a sterile scalpel. Heteroprokaryotic cell densities and cell abundance in seawater samples were determined after fixation, extraction, and staining (details in SI). Analyses were performed with a BD Acuri C6 flow cytometer (BD Biosciences, San Jose, CA, USA). Densities at the algal surface were expressed using the measured surface of each frond (details in SI and Fig. S1).

### DNA extraction, 16S rRNA gene amplification, and high throughput sequencing

For the metabarcoding approach, epiphytic cells were also collected from algal samples and rocks by gently scraping the surface with sterile scalpels [14]. Seawater (5L) was filtered using 0.2 *μ*m Millipore filters. DNA from algal and rocky surfaces was extracted using the DNeasy PowerBiofilm Kit (Qiagen, Courtaboeuf, France) according to the manufacturer’s instructions. DNA from filtered seawater was extracted using the SA-Gen protocol [45]. For all samples, the V4–V5 regions of 16S rRNA gene were targeted and amplified using 515F-Y/926R primers [46], following the PCR protocol from [47] (details in SI). Amplicons were cleaned, concentrated, quantified, and pooled at equimolar concentrations as described in [47] (details in SI). Pooled amplicons were sent to Genoscreen platform (Lille, France) for Illumina MiSeq 2 × 250 pb paired-end sequencing.

### 16S rRNA gene metabarcoding data processing and analysis

16S rRNA gene reads were processed using the FROGS workflow [48] (details in SI). An average number of 13 827 (± 10 779) 16S rRNA gene sequences per sample was obtained after filtration of sequences affiliated to chloroplasts and mitochondria (which represented an average of 37.5 ± 23.5% of total number of sequences per sample). *α*-diversity measures were estimated with Chao1 and Shannon indexes using the rarefied dataset (rarefaction performed to the minimum library size, see SI). As recommended by [49, 50], all the other analyses were conducted without rarefaction, using the dataset normalized to the total number of sequences per sample. *β*-diversity was analyzed with a non-metric multidimensional scaling (NMDS) and distance base redundancy analysis (db-RDA) using Bray-Curtis distance with “phyloseq” and “vegan” packages, respectively [51, 52]. The use of environmental constraints for db-RDA is detailed in SI. LEfSe analyses were performed to reveal specific taxa using the online tool from the Galaxy environment [53]. SIMPER analyses were performed at the genus level with the “vegan” package to identify which genera are contributing the most to the dissimilarity between samples [54]. The algal core community (ACC) of each algal sample was defined as the community spatially and temporally stable in terms of composition, and thus by keeping only OTUs occurring at least in one of each replicate. This definition was used according to [14] and prevent to fix an arbitrary percentage. The algal-enriched community (AEC) was defined by keeping only OTUs with an average relative abundance 10 times higher for algal samples compared to seawater and rocky samples.

### Extraction of surface metabolome and UPLC-ESI-MS analyses

The surface metabolome was extracted in the laboratory by dipping each frond in 5 mL of LC-MS grade methanol (Carlo Erba, Peypin, France) during 5s according to [38] and subsequent sample preparation is detailed in SI. Analyses were performed on a UHPLC-ESI-HRMS system (Dionex Ultimate 3000 rapid Separation; ThermoFisher Scientific, Illkirch, France) with an analytical core-shell reversed phase column (150 × 2.1 mm, 1.7 *μ*m, Kinetex Phenylhexyl; Phenomenex, Le Pecq, France) coupled with a QToF Impact II mass spectrometer (Bruker Daltonics, Bremen, Germany) in positive mode (details in SI).

### Metabolomics data processing, annotation, and statistical analyses

Raw UPLC-MS data were converted into netCDF files using DataAnalysis software (version 4.3, Bruker Daltonics) and processed for peak finding, integration, and alignment using the XCMS package [55] (details in SI). Data were filtered according to [56] by considering signal/noise ratio, coefficient of variation, and correlation. The resulting data matrix was log_10_-transformed, mean-centered, and normalized using the sum of the chromatographic peak areas as described in [44] and analyzed using PCA followed by partial least-square discriminant analysis (PLS-DA) allowing to reveal discriminant features according to sampling months or sites. The most discriminant features were selected according to their significance and their variable importance in projection (VIP) scores to pay attention on their annotation.

The annotation step was performed as described in [14, 44]. In brief, annotation was assessed by comparison of *m/z* value, retention time, and MS/MS spectra with those of our in-house database [57] (including purified compounds and commercial standards listed in SI) and public databases (e.g., MetLin, LipidMaps). To confirm our annotation, elucidation of the fragmentation pathways was performed and, when possible, compared to the literature. The whole dereplication procedure was facilitated using molecular networking (GNPS platform; details in SI).

### Integration of metabolomics, metabarcoding, and environmental datasets

Environmental parameters, 16S rRNA gene metabarcoding and surface metabolomics datasets were analyzed together into a global integrative approach to assess the correlations existing between each group of variables. To perform this analysis, the DIABLO approach using mixOmics R package [58] was used with the *block.splsda()* function, allowing to build a correlation network with the most correlated and discriminant features of each dataset (details in SI). The main discriminant metabolites (determined from PLS-DA, VIP scores > 2) and biomarker OTUs (determined from LEfSe, LDA scores > 3.5) found in the resulting correlation network were plotted as a function of the environmental parameters. These plots were overlaid with smoothed conditional means plots using *geom_smooth()* function (loess regression, span = 0.6).

### Statistical tests

ANOVA followed by HSD Tukey’s tests were used to evaluate the significance of variables (diversity metrics and metabolites) across the different groups of samples with “ade4” and “agricolae” R packages [59, 60]. After NMDS and PCA, differences between groups were statistically checked with two-way PERMANOVA tests combining “Site” and “Month” factors, followed by multivariate pairwise tests using “vegan” and “RVAideMemoire” packages, respectively [52, 61]. The db-RDA model was statistically validated as described in SI. PLS-DA were subjected to cross-validations tests calculated with the 6 first components, using the MetaboAnalyst tool [62].

## Results

### Environmental conditions and occurrence of *T. atomaria*

Sporophytes of *T. atomaria* were observed from February to May at S3 and S5 and from February to July at S2 and S4. According to thallus observations and length measures (Fig. S2), only one generation of sporophytes was observed for all sites except at S1, where a first generation was observed from February to April followed by a second one from May to June.

From February to July, an increase of seawater temperature from 13 to 22°C was observed for all sites, together with stable salinity and pH levels around 38 and 8, respectively (Fig. 1, Table S1). For the seawater chemical analyses, no significant differences were noticed for TN, DOC, and silicate concentrations, neither between sites nor sampling periods (ANOVA: *p* > 0.05). Significant differences were observed between months for phosphate and nutrient concentrations (ANOVA: *p* < 0.001), but not between sites (ANOVA: *p* > 0.05). Phosphate concentrations decreased from April to June with average concentrations ranging from 0.28 *μ*M (±0.07 *μ*M) to 0.11 *μ*M (±0.04 *μ*M) and then increased in July (0.25 ± 0.02 *μ*M) (Fig. 1, Table S1), while nitrate concentrations increase from June to July with average concentrations ranging from 0.34 *μ*M (±0.04 *μ*M) to 0.64 *μ*M (±0.01 *μ*M) (Table S1). Such concentrations are characteristic of mesotrophic systems. S1 was characterized by high concentrations of copper, lead, and zinc for both total and dissolved fractions, with respective values going up to 63.2, 15.2, 109.2, and 37.5, 1.7, 61.0 nM, respectively (Table S1). S5 was also characterized by a specific metal trace contamination with relative high concentrations of lead, zinc, and cadmium, especially in April (M3) with 3.7, 338.9, and 0.43 nM for the dissolved fraction (Fig. 1, Table S1).

### Epibacterial community abundance

When considering all sites together (Fig. S4A), heteroprokaryotic cell densities of algal samples appeared to be significantly lower for the two first sampling months (February and March) compared to the following ones (from April to July). From February to July, densities were ranging from 10^3^ up to 10^6^ cells.cm^-1^. When considering all sites independently (Fig. S3A), a temporal increase was observed for all sites (not significant for S5). In addition to temporal differences, sampling sites appeared also to differ (ANOVA: *p* < 0.001), with higher densities for S2 compared to S5. For seawater samples, no significant variation of cell abundance was observed neither across the different sampling months, nor the sites (Fig. S3B).

### Epibacterial diversity and community composition

**Fig. 2:**
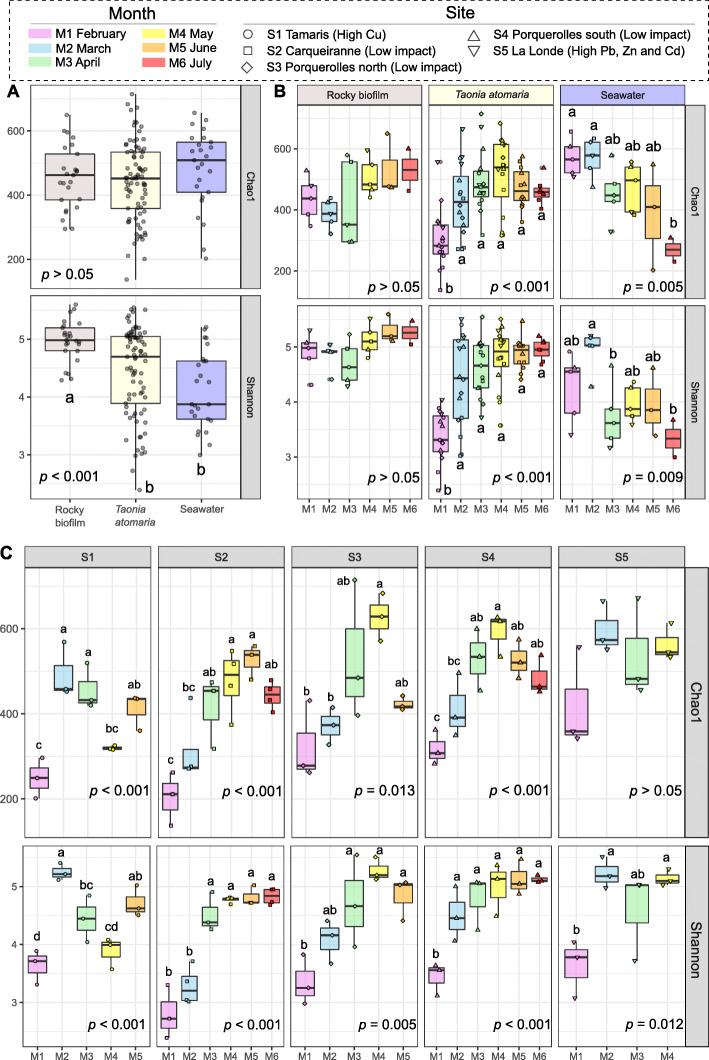
*α*-diversity metrics (Shannon and Chao1 indexes) for *T. atomaria*, rocky biofilms, and seawater samples. A Comparison between sample types for all samples, *p* values corresponded to the results of a one-way ANOVA with sample type as a factor. B Comparison between months within each sample type. C Comparison between months within each site, for algal samples. B, C *p* values corresponded to the results of a one-way ANOVA with month as factor

Overall, Shannon indexes showed significant higher values for rocky biofilms in comparison to algal and seawater samples, while Chao1 indexes did not appear significantly different (Fig. 2A). For both indexes, a temporal increase was the major tendency for algal samples, while no difference was observed according to sites (Fig. 2B and S4B). However, S1 showed a lower value in May, depicting two periods of *α*-diversity increase (Fig. 2C). For rocky samples, both indexes did not show any significant difference regardless of the month or site. For seawater samples, only the Chao1 index significantly decreased from February to July (Fig. 2B).

**Fig. 3:**
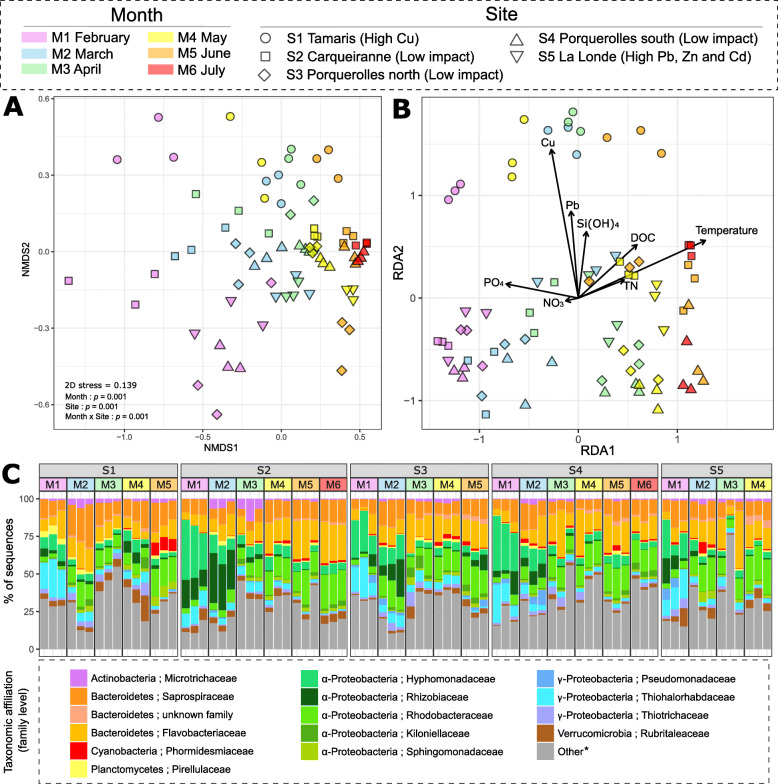
Structure and *β*-diversity of bacterial communities. **A** NMDS constructed with Bray-Curtis index showing *β*-diversity of algal samples. *p* values corresponded to results of two-way PERMANOVA using “Month” and “Site” as factors. **B** db-RDA score plot showing environmental parameters as explaining variables of the *β*-diversity of algal samples. *p* value corresponded to the result of a two-way ANOVA using “Months” and “Sites” as factors. **C** Epibacterial community composition of *T. atomaria* at the family level. ^*^“Others” correspond to unaffiliated families and families below 1%

*ß*-diversity was analyzed through NMDS analysis with a Bray-Curtis distance matrix, at first with all samples. The NMDS showed a clear significant discrimination between communities from seawater, rocky and *T. atomaria* samples (Fig. 3A, Table S2). When focused on *T. atomaria*, the NMDS (Fig. S5) mainly showed a temporal shift on the first axis with significant differences observed between each month (Table S3). Then, differences between sites were observed on the second axis, with mainly S1 which appeared as the most discriminated site (Fig. S5). All the sites were significantly different from each other, except S3 and S4 (Table S3). The db-RDA showed a similar clustering pattern as the NMDS (Fig. 3B), with differences along the first axis, mainly explained by the continuous increase of temperature, together with a decrease of PO_4_^3-^, from February to July (Fig. 1, Table S1). Differences between communities observed at S1 and those from the other sites seemed to be explained by higher copper and, to a lesser extent, lead concentrations (Fig. 3B). Singularly at S1, considering both NMDS and db-RDA analyses, communities observed in May appeared located close to those found in February and March, in relation to the second algal generation specifically observed at this site (Fig. S5 and Fig. 3B). With the NMDS constructed only with rocky samples, no significant clustering appeared neither between months, nor sites (Fig. S6A, Table S4), and no significant effect of trace metal has been observed using a db-RDA model (PERMANOVA: *p* > 0.05). For seawater samples, a significant clustering only appeared between months whatever the site (Fig. S6B, Table S5).

The structure of prokaryotic communities differed significantly between algal surface and seawater. This observation was mainly due to the dominance in seawater of specific genera such as *Synechococcus*, *Litorimicrobium*, an unknown genus of *Pirellulaceae*, and *Planktotalea* contributing to 6.8, 2.9, 2.3, and 1.4% of the total dissimilarity, respectively (Table S6). Several discriminant taxa found in a higher abundance in rocky samples were identified as members of the genera *Loktanella* and *Lewinella* and the cyanobacterial order *Nostocales*, mainly represented by the genus *Pleurocapsa* (Fig. S7). These three genera, together with *Maritimonas* and *Aquimarina*, contributed for 1.9, 1.2, 0.8, 1.4, and 1.2% of the total dissimilarity within algal samples, respectively (Table S7). In contrast, several taxa were specific to algal samples, including *Litorimonas*, *Granulosicoccus*, *Algitalea*, and *Nitratireductor* at the genus level, and *Hyphomonadaceae*, *Thiohalorhabdaceae*, *Saprospiraceae*, and *Rhizobiaceae* at the family level (Fig. S7).

Among algal samples, the epibacterial composition at the family level (Fig. 3C) was always similar within replicates for a same sampling site and a same month, except for samples from S5 at M3 for which one replicate was characterized by a higher abundance of unaffiliated families. A clear temporal shift appeared whatever the site with the main occurrence of specific families, such as *Hyphonomonadaceae* and *Thiohalorhabdaceae*, in February (Fig. 3C). These two families, mainly represented by the genera *Litorimonas* and *Granulosicococcus*, respectively, appeared as biomarkers of this month whatever the site (Fig. S8). From February to July, the average relative proportions of these families decreased from 22.3 to 4.9% and from 15.4 to 1.1%, respectively. In contrast, a continuous temporal increase was observed for the families *Rhodobacteraceae* and *Flavobacteriaceae* (from 3.5 to 18.0% and 7.8 to 18.5%, respectively). Through the LEfSe analysis, these two families were also specific taxa of July (Fig. S8). Since the temporal discrimination was preponderant in the clustering of the whole set of algal samples, discriminant analyses according to sites were conducted, firstly with all months, and then month by month (not shown). Whatever the month, several taxa were found as site biomarkers such as *Rubritaea*, *Roseibacillus*, *Ornithococcus*, *Clostridiales*, and *Micrococcales* for S1, *Ekhidna* for S2, *Persicirhabdus* for S3, *Sphingobacteriales* for S4, and *Nostocaceae* for S5 (Fig. S9). Considering each month independently, *Rubritalea* appeared recurrently discriminant for S1, from April to June (LDA > 3.5).

Both relative percentages of OTUs and sequences of the ACC showed a significant decrease mainly from February to May (Fig. S4C and S10A). Percentages of OTUs and sequences of the AEC showed a similar tendency to the ACC with a significant decrease observed from February to March (Fig. S4D and S10B). As for the total community, *Hyphomonadacaceae* and *Thiohalorhabdaceae* were found as the major families of the early ACC and AEC and appeared to decline from February to April, while *Rhodobacteraceae* and *Flavobacteriaceae* increased after February (Fig. S10). With the db-RDA plots representing also OTU positions (Fig. S11), four of the five most abundant core OTUs were associated to February and March samples, negatively correlated to the temperature increase, and affiliated to *Litorimonas* (Hyphomonadaceae), *Granulosicoccus* (*Thiohalorbdaceae*), and *Nitratireductor* (*Rhizobiaceae*). In contrast, another core OTU affiliated to *Algitalea* (*Flavobacteriaceae*) and associated with samples going from May to July was positively and negatively correlated with the temperature and PO_4_^3-^ increases, respectively. Interestingly, no core OTUs were associated to high levels of trace metal concentrations (Fig. S11).

### Surface metabolome profiling

**Fig. 4:**
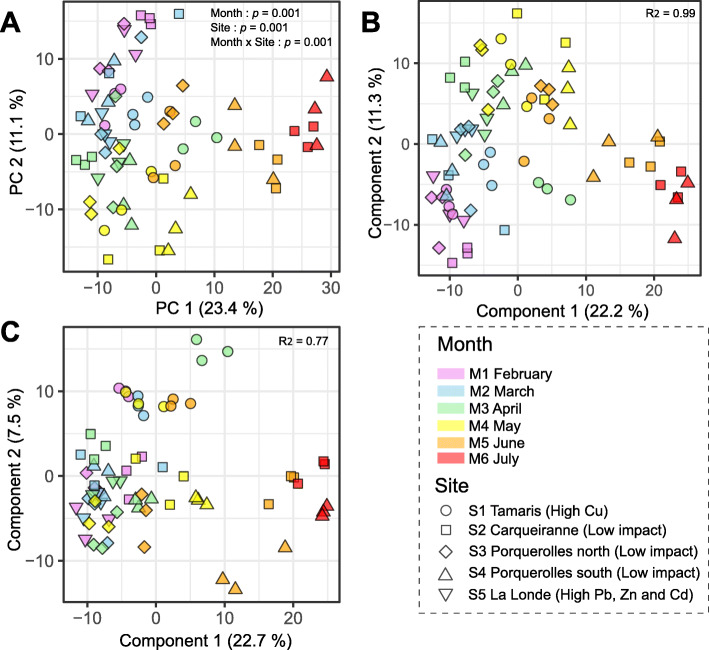
Diversity surface metabolome profiles assessed through LC-(+)-ESI-MS metabolomics. Score plots of **A** PCA and PLS-DA constructed either with months (**B**) or sites (**C**) as supervised groups. *p* values corresponded to results of a two-way PERMANOVA using “Month” and “Site” as factors

The surface metabolomics dataset (422 *m/z* features) was first analyzed by PCA (Fig. 4A) and showed a clear temporal shift according to each month (Table S8). Statistical differences between sites were observed, except between S2 and S4 (Table S8). Component 1 (23% of the total variance) showed a metabolomic shift from May to July whereas component 2 (11% of the total variance) mainly explained the metabolomic shift from February to May. Two PLS-DA models were constructed using either months or sites as supervised groups. The PLS-DA model discriminating months (Fig. 4B) showed a score plot highly similar to the pattern obtained with the PCA. The second PLS-DA model discriminating sites showed a clear difference between S1 and the other sampling sites (Fig. 4C).

### Surface compound characterization and dynamics

Molecular networking gave an overview of the surface metabolome by gathering the main chemical families (Fig. S12). Clusters A and F were composed by terpenes (mainly diterpenes and sesquiterpenes). Clusters B and D revealed di- and monoacylglycerylhydroxymethyl-*N*,*N*,*N*-trimethyl-*β*-alanines (DGTAs and *lyso*-DGTAs), respectively, while cluster C gathered monogalactosyldiacylglycerols (MGDGs) and diacylglycerols (DGs). Cluster E was characterized by di- and monoacylglyceryl-3-*O*-carboxy-(hydroxymethyl)-choline (DGCCs and *lyso*-DGCCs) and cluster G by pheophytin a and other chlorophyll derivatives.

**Fig. 5:**
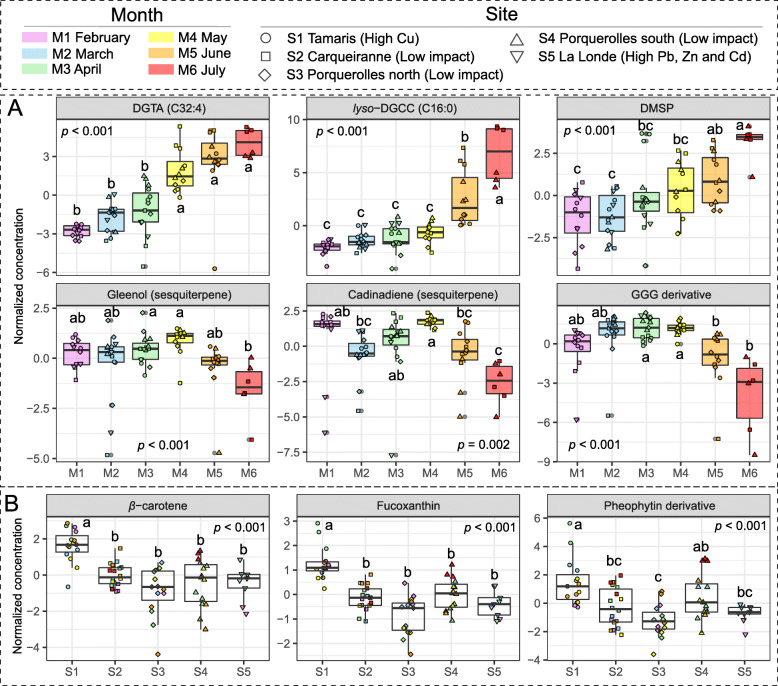
Normalized concentrations of significant and discriminant surface metabolites. **A** Discriminant metabolites according to temporal differences. *p* values corresponded to the results of a one-way ANOVA using “Month” as factor. **B** Discriminant metabolites according to spatial differences. *p* values corresponded to the results of a one-way ANOVA using “Site” as factor

Furthermore, a focus was made on the annotation of discriminant features (VIP score > 2), differentially expressed among months or sites (Table S9 and S10). Among the 15 most discriminant features according to the different months, 7 compounds were identified as DGTAs and showed the same pattern with increasing concentrations during the seasonal monitoring (Fig. 5A, Table S9). Several other compounds showed similar seasonal variations, in particular *lyso*-DGCC (C16:0), *lyso*-phosphatidylcholine (*lyso*-PC, C20:5), and DMSP (Fig. 5A, Table S9). Despite lower VIP scores, sesquiterpenes (such as gleenol and cadina-4(14),5-diene) together with a geranylgeranylglycerol derivative showed a common pattern characterized by a significant decrease from May to July, while no clear change was observed before (Fig. 5A).

When focusing on features differentially expressed according to sampling sites (VIP score > 2), all selected metabolites, including DG (C20:5/C16:4), pigments such as fucoxanthin, *β*-carotene, and a pheophytin derivative (Fig. 5B, Table S10), were identified as biomarkers of S1. These compounds were found in significantly higher concentrations at S1 compared to the other sites, except for the pheophytin derivative at S4 (Fig. 5B, Table S10).

### Multi-omics network analysis

**Fig. 6:**
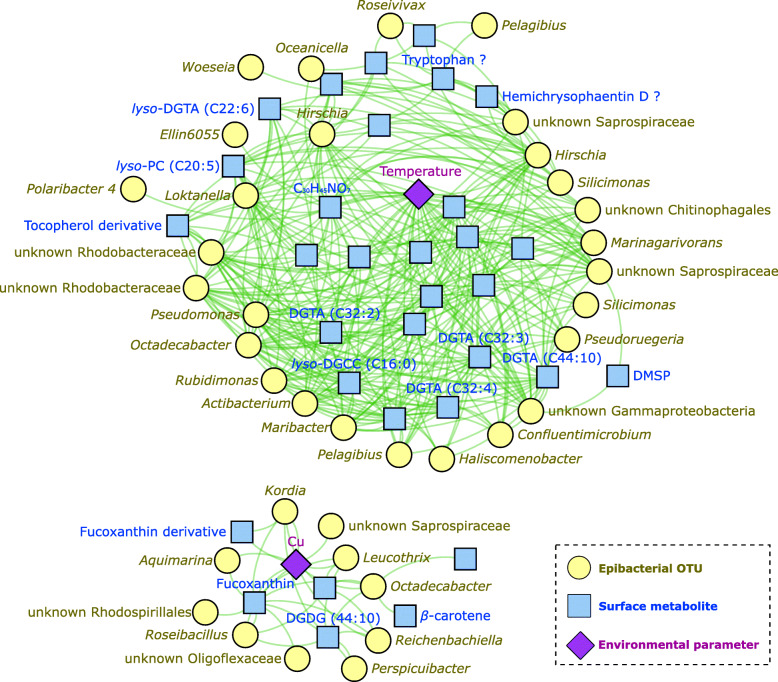
Spatiotemporal correlation network (multi-block sPLS-DA, DIABLO analysis) of the surface metabolome, epibacterial community, and environmental variables. Using a sparse method, the network was constructed with an optimal number of variables according to the tuning procedure, which corresponded to a total of 32 metabolites, 37 OTUs, and 2 environmental parameters with only positive correlations above 0.7 and negative correlations below −0.7

A multi-omics-based network (Fig. 6) revealed correlations between the most discriminant features from each dataset (OTUs from 16S rRNA gene metabarcoding, surface metabolites from metabolomics, and environmental data). The whole network showed two clusters with only positive correlations. Temperature and copper concentrations were the only two environmental parameters showing correlations with features of other datasets, one in each cluster.

The first cluster, associated to the seawater temperature, gathered variables involved in the temporal variations. This cluster was composed by characteristic variables of the summer period, such as surface metabolites corresponding to several DGTAs, *lyso*-DGCC (C16:0) and DMSP. This latter is one of the most discriminant metabolites increasing with time (Table S9), and its relative concentration was investigated in more details, as a function of the temperature changes, showing a non-parametric temporal increase with this environmental parameter (Fig. S13). OTUs positively associated with this cluster were also found with increasing abundances during the seasonal monitoring and were mainly affiliated to the family *Rhodobacteraceae* (e.g., genera *Loktanella*, *Pseudoruegeria*, *Silicimonas*, and *Octadecabacter*). As for the DMSP, these OTUs also showed a temporal increase together with the temperature (Fig. S13).

The second cluster was composed by features specific to S1, copper concentrations being the central environmental parameter. Within this cluster, fucoxanthin, a fucoxanthin derivative, and DGDG (C44:10) were positively correlated to copper concentrations. Moreover, this cluster gathered two identified VIPs specific of S1: fucoxanthin and *β*-carotene (Table S10). Normalized concentrations of these two chemomarkers were investigated as a function of copper concentration, suggesting a non-parametric regression, with an increase up to 15 nM, followed by a plateau from 20 nM to 30 nM (Fig. S14). Two OTUs of this cluster were affiliated as biomarkers genera specific of S1: *Roseibacillus* (*Rubritaleaceae*) and *Kordia* (*Flavobacteriaceae*) (Fig. S9). This two OTUs were also positively correlated to copper and the metabolites previously mentioned. The relative percentages of these two OTUs were investigated as a function of the copper concentration and also suggested a non-parametric regression with a peak reached at 15 nM of copper (Fig. S14).

## Discussion

As the holobiont concept required to consider both hosts and their associated microbiota in integrative studies, a multi-omics approach coupling metabolome and microbiota analyses at the surface of *T. atomaria* was conducted on five environmentally different sites to investigate how environmental parameters may control the host-microbiota relationships. Regarding the metabarcoding approach, low variabilities between replicates were observed, whether for the *α*-diversity, or the community structure at the family and the OTUs level, suggesting that the community assembly at the algal surface might not follow a fully stochastic process.

*β*-diversity from surface communities on *T. atomaria* appeared clearly dissimilar from those from neighboring rocky surfaces or seawater. This indicated for the first time for this Mediterranean seaweed that its epiphytic microbiota was highly host-specific. Moreover, the Shannon index was lower for algal samples compared to rocky biofilms irrespective of time or site, which also supports the hypothesis of a control of the surface microbiota by the algal host. The *β*-diversity dissimilarity between algal epiphytic communities and epilithic ones have also been observed in few studies conducted within other environments with Canadian kelps and *Mastocarpus* spp. [63, 64] or *Fucus vesiculosus* from the Baltic sea [16]. Additional mechanisms of selection must be involved, including the role of algal physical properties (e.g., surface structuration and rigidity), but results from this integrated multi-omics analysis indicated that metabolites expressed at the algal surface, together with environmental constraints, should be viewed as the main factors shaping the structure and the physiology of the epiphytic microbiota, consequently influencing the whole dynamics of interactions in the holobiont. In terms of methodology, the use of a “mock community” could have allowed to bring more confidence in the affiliation of the bacterial taxa. However, building a representative and reliable “mock community” composed by major algal epiphytic strains would be challenging.

### A winter, low diversified, and host-specific pioneer community

The pioneer microbiota was defined in this study as the part of the algal epibacterial community involved in the colonization of *T. atomaria* surface at its early stage (in February, M1), when the thalli length reached around 4 cm (±0.2 cm) and the algal individuals are approximately 20 days old (Fig. S2). This pioneer community was mainly characterized by the occurrence of *Hyphomonadaceae* and *Thiohalorhabdaceae* whatever the site, as previously reported at S1 and S2 [14, 44]. *Hyphomonadaceae*, mainly represented by the genus *Litorimonas*, appeared as a major contributor of the core and host-enriched microbial consortia associated to the algal surface. The core community (defined here by keeping only OTUs occurring at least in one of each replicate for each condition) is generally considered as a stable community composed by key taxa displaying significant functions for the host fitness within plants [65], and which could be considered characteristic of healthy seaweeds [66]. The *Hyphomonadaceae* family was already described on eukaryotic surfaces [67], notably *Litorimonas cladophorae* isolated from the Chlorophyta *Cladophora stimpsoni* and *Algimonas porphyrae* from the Rhodophyta *Porphyra yezoensis* [68, 69]. Moreover, a recent study showed that two OTUs affiliated to the genus *Litorimonas* were also reported as core OTUs of the Rhodophyta *Mastocarpus* spp. and showed a higher abundance on these algal surfaces compared to seawater and rocks [64]. This observation strengthened the idea that *Hyphomonadaceae*, and especially the genus *Litorimonas*, are particularly well-adapted to the macroalgal niche. This high specificity was hypothesized to be linked to several functions including the use of algal exudates or dioxygen produced by the host [67].

Moreover, *Hyphomonadaceae* are known to produce a polar holdfast which facilitates the surface anchoring [70]. In the case of *Hyphomonas* strain VP-6, this holdfast synthesizes specific EPS implied in the first step of the cell attachment [71]. These functions described in the literature could provide selective advantages for the attachment of pioneer taxa at the surface of uncolonized young algal tissues. Further “-omics” or qPCR studies focusing on functions associated to chemotaxis, secretion, or adhesion systems, and more broadly biofilm formation could provide relevant insights, considering that such features could appear clearly essential for the establishment of pioneer taxa which generally required the production of motility proteins, surface chemo- and mechano-sensors, and EPS [70]. During the two following months, *Hyphomonadaceae*, and notably *Litorimonas*, showed a decrease in relative abundance. Conversely, cell densities and *α*-diversities increased from February to April, indicating the maturation of biofilms at the algal surface after the early colonization process of pioneer, low diversified, core, and algal preferential taxa.

### Temperature as a major temporal shaping factor

The occurrence of *Hyphomonadaceae* at the early algal stage could have been linked to low temperatures, but such bacteria are probably more psychrotrophs than psychrophyles (i.e., more tolerant than adapted, with a likely growth at high temperatures), since they seemed to reappear as a major family when a second generation of thalli started at S1 in May, when seawater temperature is high. Similarly, the beginning of this new generation of young sporophytes in May at S1 could also explained lower *α*-diversity metrics and higher percentages of AEC and ACC associated to the pioneer community. Indeed, as observed in [44], younger colonized surfaces of *Taonia* are characterized by a less diverse and abundant communities than older ones, in line with the epibacterial diversification associated to the algal growth. An experimental study conducted through controlled laboratory conditions could be an interesting approach to better decipher if the decrease of the algal pioneer and specific epibacterial communities, together with the increase of *α*-diversity, could be linked to algal age or to the temperature increase.

In contrast, the family Rhodobacteraceae constitutes a major taxon increasing during the entire monitoring period. This family was mainly represented by genera of the Roseobacter, such as *Octadecabacter*, *Litoreibacter*, *Sulfitobacter*, *Loktanella*, *Pseudoruegeria*, and *Silicimonas*, which were distinct from those dominating in the surrounding seawater (e.g., *Planktotalea* and *Litorimicrobium*). Among this family, OTUs affiliated to *Octadecabacter*,*Silicimonas*, *Loktanella*, and *Pseudoruegeria* were found positively correlated to the seawater temperature. The *Rhodobacteraceae* family was already described at the surface of many macroalgae and several taxa within this family were recurrently associated to high temperature conditions [27, 72–76]. Especially, in the case of *D. pulchra*, the temperature was associated to the development of several pathogens from the *Roseobacter* group, such as *Nautella italica* [20, 74]. Moreover, other taxa of the *Roseobacter* group have been described to be attracted by DMSP and able to use it as carbon and sulfur sources [77–80]. DMSP constitutes an important osmolyte, synthetized by many microalgae but also seaweed [81–83], which is involved in various seaweed-bacterial interactions [9, 10, 84, 85]. As for [14], metabolomics showed here that DMSP also increased from February to July and appeared correlated with seawater temperature. The increasing production of DMSP at the algal surface could allow a better development of an adapted community specialized in the use of DMSP, notably for several taxa of the *Roseobacter* group showing DMSP catabolic pathways in their genomes (e.g., *Octadecabacter antarticus*) [78].

In contrast with *T. atomaria*, the DMSP content of Chlorophyta *Codium fragile* was found to decrease with seawater temperature but to increase with sunlight intensity [82]. Concurrently to temperature, irradiance increased from February to July along the French Mediterranean coast. Thus, irradiance could also explain the seasonal variations of DMSP at the surface of *T. atomaria* rather than temperature itself. This hypothesis was strengthened by other studies revealing positive correlations between the DMSP content of five Chlorophyta from Antarctica and day-length and sunlight intensity [81, 86]. Similar observations were also noticed in phytoplankton species (e.g., *Phaeocystis* sp.), and the related emission of dimethylsulfide in the atmosphere was even proposed to participate to climatic changes (see the CLAW hypothesis, [80, 83, 87, 88]). Considering that biofilms at the seaweed surface are more mature and that irradiance is higher during the summer period, a higher density of DMSP-producing microorganisms (e.g., diatoms, dinoflagellates, and coccolithophores, but also some bacteria from the *Alphaproteobacteria*) may be expected [89, 90], substantially participating to the DMSP production at the algal surface. The relative importance of temperature and irradiance is challenging to investigate with in situ approaches due to their covariations. An experimental approach based on controlled cross-conditions of these two factors needs to be considered to understand their respective effects on DMSP production at the surface of *T. atomaria*, and more generally on the overall holobiont dynamics.

Sesquiterpenes and a geranylgeranylglycerol derivative observed at the surface of *T. atomaria* showed a significant decrease after May. Among these terpenes, gleenol and geranylgeranylglycerol had been previously described for their anti-adhesion activities against marine bacteria [38]. Sesquiterpenes are also known as volatile chemical defenses against pathogens in the case of terrestrial plants [91, 92]. In a similar way to *D. pulchra* with halogenated furanones [13], the late decrease of chemical defenses could be linked to the reduction of the host fitness caused by the stress of increasing temperature, irradiance, fouling pressure, or also by the aging process. Consequently, the lower level of chemical defenses expressed notably in July could promote the development of opportunistic epibacterial pathogens, but also specific macrofoulers. Indeed, for some *T. atomaria* individuals collected in July, the colonization of encrusting coralline algae was occasionally observed on the thalli together with bleached apical tips (data not shown).

### Trace metal contamination plays locally a key role on the holobiont fitness

For both surface metabolomics and microbiota profiles, a clear discrimination of the samples in relation with trace metal contamination was reported. In the case of S1, a high enrichment factor for copper, and to a lesser extend for zinc and lead, was observed compared to the uncontaminated sites S2–S4, or the occasionally contaminated site S5, and reflected the high level of anthropogenic pressures of the bay of Toulon [42, 43]. Furthermore, contamination profile at S5 appeared specific to April with high concentrations of lead, zinc, and cadmium (but not copper) in both dissolved and total fractions but without clear effects on the epibacterial community. The strong Mediterranean rain events occurring in March and April (notably on March 25, 2017, April 02, 2017, and April 26, 2017) could have generated a water spill from the river close to S5 (Le Maravenne) and then a subsequent resuspension of metals in the coastal sea. In particular, lead is known to be easily remobilized from contaminated particles in coastal water column [93, 94]. The specific metabolomics and microbiota profiles at S1 could probably be linked to high metal contaminations, especially in copper, as dissimilar biofilm communities were already reported between contaminated vs uncontaminated areas in the bay of Toulon [95]. Considering the relative role of metals, lead was shown to have limited effect compared to copper on microbial communities during a mesocosms experiment mimicking the environmental conditions of the bay of Toulon [96].

Several epibacterial taxa appeared specific to the copper-rich site S1, notably the *Kordia* genus (*Flavobacteriaceae* family), and the *Rubritaleaeceae* family (*Verrucomicrobia* phylum), mainly represented by two OTUs of the genera *Rubritalea* and *Roseibacillus*. Interestingly, *Rubritalea* has been already observed as a specific epiphytic taxon of the Chlorophyta *Ulva* spp. in copper-rich environment [97]. Another study shows that *Rubritalea squalenifaciens* biosynthesizes antioxidative carotenoids to prevent lipid peroxidation [98]. Despite bioassays conducted with higher copper concentrations (from 0 to 8 mM), copper stress is actually known to quickly induce peroxidation of membrane lipids of bacteria [99] and, similarly to algae, production of carotenoids could constitute a selective advantage against related oxidative stress conditions [100].

However, hypotheses of direct or indirect effects of copper on surface community selection remain difficult to address with field analyses. For example, indirect effects through copper toxicity to phytoplankton and consecutive specific organic matter release have been suggested to select copiotroph taxa [43]. In the case of epibacterial communities, host surface metabolites could also play a major role. Among discriminant surface metabolites in these copper-rich conditions, the overexpression of photosynthetic pigments, fucoxanthin, pheophytin derivatives, and *β*-carotene was observed. These results were in accordance with previous studies showing that several macroalgae exposed to similar copper concentrations than those found at S1 and increased their production of pigments (carotenoids and chlorophylls) as an adaptation strategy in response to photo-inhibition and reactive oxygen species (ROS) production induced by copper [101–103]. For several macroalgae submitted to high copper stress, electron transport rate have been found to be inhibited [104] and Cu^2+^ also competes with Mg^2+^ located in the chlorin ring of chlorophyll a, inactivating the associated light-harvesting function and leading to high toxicity [105]. Similarly, *T. atomaria* could express, under copper-rich conditions, higher concentrations of pigments to prevent photo-inhibition, and, more specifically, carotenoids known for their antioxidant properties to avoid ROS-induced damage. However, these photopigments were not linearly expressed as a function of the copper concentration (with a maximum concentration reached in the case of a copper concentration of 20 nM), suggesting a limit in the antioxidative response of the seaweed. The oxidative stress promoted by such copper contamination has been also shown as a major factor of changes of the lipidome for several seaweed [106–108]. Here, in addition to photosynthetic pigments, high copper concentrations could be the cause of the changes observed in the algal surface lipidome, especially with a higher production of DG (C20:5/C16:4). In addition, as no clear relationship could be conversely established between copper concentrations and communities from rocky biofilms for the sampled sites, the indirect effect of copper through its impact on the host could be preferential in the selection of a specific community at Tamaris (S1).

Consequently, the shift of the surface algal metabolome potentially induced by copper stress, with notably a higher expression of fucoxanthin, could be a key parameter involved in the spatial dissimilarities of the epiphytic microbiota. In the case of *Dictyota* sp., but also for the more phylogenetically distant *F. vesiculosus*, this xanthophyll implied in the photosynthesis process has been identified as an inhibitor of the bacterial adhesion [84, 109]. Moreover, fucoxanthin was observed as an inhibitor of bacterial settlement in field studies for *F. vesiculosus* and consequently considered as a part of its chemical defenses [110]. However, no clear effect was observed on the microbial community composition at the surface of *F. vesiculosus* [110], and the potential role of this compound in the selection of specific epiphytic communities needs to be more deeply investigated [2].

## Conclusion

In conclusion, we found that (i) epibacterial communities were highly specific to *T. atomaria*, especially in the case of winter and low diversified pioneer bacterial colonizers, (ii) epibacterial densities and *α*-diversity measures increased over months and witnessed the attachment of new colonizers reducing the proportion of core and algal preferential taxa, (iii) strong spatio-temporal co-variations occurred between surface metabolites and epibacterial communities suggesting a whole holobiont dynamics, and (iv) the temperature increase and trace metal contaminations constituted major direct and/or indirect environmental factors shaping seaweed-epibacterial interactions.

This study constituted the first multi-omics approach coupling metabolomics and metabarcoding analyses to investigate the dynamics of seaweed-epibacteria interactions in connection with environmental parameters. Especially, in the case of trace metal contamination, whose impact has been rarely considered, this work brought new insights to decipher anthropogenic effects on marine coastal ecosystems.

## Supplementary Information

**Additional file 1: Table S1** Environmental parameters and sampling dates (Part 1: from February to April 2017). For trace metals, (d) and (t) indicated dissolved and total acid leachable fractions, respectively. n.d.: not determined. **Table S2** Multivariate pairwise results (*p* values) examining differences between “sample types” for the 16S rRNA gene dataset with all samples (999 permutations). **Table S3** Multivariate pairwise results (*p* values) examining differences between “months” and between “sites” for the 16S rRNA gene dataset with *Taonia atomaria* samples (999 permutations). **Table S4** Multivariate pairwise results (*p* values) examining differences between “months” and between “sites” for the 16S rRNA gene dataset with rocky biofilm samples (999 permutations). **Table S5** Multivariate pairwise results (*p* values) examining differences between “months” and between “sites” for the 16S rRNA gene dataset with seawater samples (999 permutations). **Table S6** SIMPER results of the most contributing genera to the dissimilarities between seawater and algal samples. Only the first 50% of the cumulative contribution is shown. *p* values were calculated with a permutation test constructed with 999 permutations and corresponded to the probability of getting a larger or equal average contribution in random permutation of the group factor. Cum. sum corresponded to the ordered cumulative contribution. **Table S7** SIMPER results of the most contributing genera to the dissimilarities between rocky biofilm samples and algal samples. Only the first 50% of the cumulative contribution is showed. *p* values were calculated with a permutation test constructed with 999 permutations and corresponded to the probability of getting a larger or equal average contribution in random permutation of the group factor. Cum. sum corresponded to the ordered cumulative contribution. **Table S8** Multivariate pairwise results (*p* values) examining differences between “months” and between “sites” for the LC-(+)-ESI-MS metabolomics dataset (999 permutations). **Table S9** List of biomarkers (VIP score > 2) identified by LC-HRMS and involved in the discrimination between months within surface extracts of *T. atomaria* (Part 1). *P* values corresponded to results of one-way ANOVA tests using “Month” as factor. Color codes corresponded to mean normalized concentrations (see Part2: Table S12). List of biomarkers (VIP score > 2) identified by LC-HRMS and involved in the discrimination between months within surface extracts of *T. atomaria* (Part 2). *P* values corresponded to results of one-way ANOVA tests using “Month” as factor. Color codes corresponded to mean normalized concentrations. **Table S10** List of biomarkers (VIP score > 2) identified by LC-HRMS and involved in the discrimination between sites within surface extracts of *T. atomaria*. *P* values corresponded to results of one-way ANOVA tests using “Sites” as factor. Color codes corresponded to mean normalized concentrations. **Figure S1** Discrimination of heterotrophic prokaryotes by flow cytometry. A successive 3-steps workflow was used. A: Samples were first screened for the presence of potential doublets or aggregates. Sample dilution was eventually adjusted in order to keep doublets below 5% of the total signal. B: Particles showing a red fluorescence (FL3) were excluded in order to keep only strict heterotrophs (*i.e.* presenting only the SYBR green-induced fluorescence). C: High side scatter signal harboring particles were excluded in order to enumerate only prokaryotes. **Figure S2** Variations of thalli length (cm) conducted with all algal samples collected during the study and pictures of thalli collected at S1 (one replicate per month). **Figure S3** Variations of cells densities at the surface of *T. atomaria* for each site (A) and heteroprokaryotic cell abundances in seawater samples (B). *p* values corresponded to results of one-way ANOVA using “Month” as the factor. **Figure S4** Spatiotemporal dynamics of prokaryotic cell density (A), ***α***-diversity (B), percentage of algal-core (C) and algal-enriched (D) taxa at the surface of *T. atomaria*. *p* values corresponded to results of one-way ANOVA using “Month” as factor. **Figure S5** NMDS (Bray-Curtis index) showing prokaryotic *β*-diversity algal samples. **Figure S6** NMDS (Bray-Curtis index) showing prokaryotic *β*-diversity of rocky biofilm (A) and seawater (B) samples. *p* values corresponded to results of one-way PERMANOVA using whether “Month” or “Site” as factors. **Figure S7** Cladogram obtained from the LEfSe analysis built with the 16S rRNA gene dataset of all samples and revealing discriminant prokaryotic taxa specific to *T. atomaria*, to rocky biofilms and to seawater samples (LDA threshold set to 4). **Figure S8** Cladogram obtained from the LEfSe analysis built with the 16S rRNA gene dataset of *T. atomaria* samples and revealing discriminant epibacterial taxa specific to each sampling month (LDA threshold set to 4.5). **Figure S9** Cladogram obtained from the LEfSe analysis built with the 16S rRNA gene dataset of *T. atomaria* samples and revealing discriminant epibacterial taxa specific to each sampling site (LDA threshold set to 3.5). **Figure S10** Percentage of core community sequences (A) and algal-enriched community sequences (B). *: “Other” corresponded to unaffiliated families and families below 1%. **Figure S11** Plots from the db-RDA scoring (A) environmental parameters together with algal samples (identical to Fig. 3B), and (B) environmental parameters together with OTUs represented by dashed circles. Dashed circles size was proportional to their relative percentage within the whole algal dataset. Dashed circles filled in pink corresponded to core OTUs, while those filled in dark blue were not affiliated as core members. The name of the 5 main core OTUs were annotated above their corresponding dashed circles. **Figure S12** GNPS molecular network built with LC-ESI-(+)-MS/MS data. Levels of annotations were attributed according to Schymanski et al., 2014. **Abbreviations: DGTA: diacylglycerylhydroxymethyl-N,N,N-trimethyl-b-alanine, DGCC: monoacylglyceryl-3-O-carboxy-(hydroxymethyl)-choline, DG: diacylglycerol, MG: monoacylglycerol, GGG: geranylgeranylglycerol, DGDG : digalactosyldiacylglycerol, MGDG : monogalactosyldiacylglycerol. Figure S13** Scatter plots showing the normalized concentration of DMSP, and the relative abundances of OTUs from the correlation network affiliated to Rhodobacteraceae (Figure S5), as a function of the temperature. Scatter plots were overlayed with smoothed conditional means plots using *geom_smooth()* function (loess regression, span = 0.6). **Figure S14** Scatter plots showing the normalized concentration of fucoxanthin and β-carotene, and the relative abundances of OTUs from the correlation network affiliated to *Kordia* and *Roseibacillus* (Figure S5), as a function of the dissolved copper concentration. Scatter plots were overlayed with smoothed conditional means plots using *geom_smooth()* function (loess regression, span = 0.6).

## Data Availability

Sequences data were deposited and are publicly available in the NCBI Sequences Read Archive (SRA) under the BioProject ID PRJNA639819, accession number. Raw data for LC-ESI-(+)-MS/MS experiments were deposited and are publicly available in the MassIVE platform under the IDs MSV000082277. Cytometry raw data are available on request from the authors.

## Abbreviations

ACC: Algal core community
AEC: Algal enriched community
ANOVA: Analysis of variance
db-RDA: Distance based-redundancy analysis
DG: Diacylglycerol
DGCC: Diacylglyceryl-3-*O*-carboxy-(hydroxymethyl)-choline
DGTA: Diacylglycerylhydroxymethyl-*N*,*N*,*N*-trimethyl-*β*-alanine
DIABLO: Data integration analysis for biomarker discovery using latent variable approaches for omics studies
DMSP: Dimethylsulfoniopropionate
EPS: Extracellular polymeric substance
FROGS: Find rapidly OTUs with galaxy solution
MGDG: Monogalactosyldiacylglycerol
NMDS: Non-metric multidimensional scaling
NW: North-west
OTU: Operational taxonomic unit
PC: Phosphatidylcholine
PCA: Principal component analysis
PERMANOVA: Permutational analysis of variance
PLS-DA: Partial least-square discriminant analysis
ROS: Reactive oxygen species
SIMPER: Similarity percentages
VIP: Variable importance in projection
