# Supplementary material for: Integration of spatio-temporal variations of surface metabolomes and epibacterial communities highlights the importance of copper stress as a major factor shaping host-microbiota interactions within a Mediterranean seaweed holobiont
Source: Microbiome. 2021 Oct 12;9:201. doi: 10.1186/s40168-021-01124-8 (PMC8507236; doi:10.1186/s40168-021-01124-8)
Supplement: Supplementary file 2 — Additional file 1: Table S1 Environmental parameters and sampling dates (Part 1: from February to April 2017). For trace metals, (d) and (t) indicated dissolved and total acid leachable fractions, respectively. n.d.: not determined. Table S2 Multivariate pairwise results (p values) examining differences between “sample types” for the 16S rRNA gene dataset with all samples (999 permutations). Table S3 Multivariate pairwise results (p values) examining differences between “months” and between “sites” for the 16S rRNA gene dataset with Taonia atomaria samples (999 permutations). Table S4 Multivariate pairwise results (p values) examining differences between “months” and between “sites” for the 16S rRNA gene dataset with rocky biofilm samples (999 permutations). Table S5 Multivariate pairwise results (p values) examining differences between “months” and between “sites” for the 16S rRNA gene dataset with seawater samples (999 permutations). Table S6 SIMPER results of the most contributing genera to the dissimilarities between seawater and algal samples. Only the first 50% of the cumulative contribution is shown. p values were calculated with a permutation test constructed with 999 permutations and corresponded to the probability of getting a larger or equal average contribution in random permutation of the group factor. Cum. sum corresponded to the ordered cumulative contribution. Table S7 SIMPER results of the most contributing genera to the dissimilarities between rocky biofilm samples and algal samples. Only the first 50% of the cumulative contribution is showed. p values were calculated with a permutation test constructed with 999 permutations and corresponded to the probability of getting a larger or equal average contribution in random permutation of the group factor. Cum. sum corresponded to the ordered cumulative contribution. Table S8 Multivariate pairwise results (p values) examining differences between “months” and between “sites” for the LC-(+)-ESI-MS metabol [file 40168_2021_1124_MOESM2_ESM.docx]

# Supplementary information for

**Integration of spatio-temporal variations of surface metabolomes and epibacterial communities highlights the importance of copper stress as a major factor shaping host-microbiota interactions within a Mediterranean seaweed holobiont.**

Benoit Paix^1^, Nicolas Layglon^2^, Christophe Le Poupon^2^, Sébastien D’Onofrio^2^, Benjamin Misson^2^, Cédric Garnier^2^, Gérald Culioli^1,^^[[1]](#footnote-1)*^ and Jean-François Briand^1*^

*^1^Université de Toulon, Laboratoire MAPIEM, EA 4323, Toulon, France*

*^2^Université de Toulon, Aix Marseille Université, CNRS, IRD, Mediterranean Institute of Oceanography (MIO), UM110, France*

*Corresponding authors: [briand@univ-tln.fr](mailto:briand@univ-tln.fr), [gerald.culioli@univ-avignon.fr](mailto:gerald.culioli@univ-avignon.fr)

# Supplementary information for Material and Methods

## Supplementary information for sampling

Thalli of *Taonia atomaria* were collected by hand, by carefully detaching the holdfast from rocky substrates, using nitrile gloves to avoid DNA contaminations on algal surface. The GPS coordinates are 43°05'35.0"N 5°54'31.0"E for S1; 43°05'12.2"N 6°05'02.1"E for S2; 43°00'16.1"N 6°11'21.9"E for S3; 42°59'41.7"N 6°10'53.3"E for S4; and 43°07'13.7"N 6°16'00.3"E for S5. Sampling dates are detailed in Table S1. No thalli of *T. atomaria* were observed on the five sampling sites after July. Rock and seawater (5L) samples were collected nearby algae samples. Rock samples were chosen haphazardly, and it resulted in various types of substrate.

In addition, surface seawater was collected in 1L-fluorinated ethylene propylene (FEP) bottles (Nalgene) previously cleaned with 10% HNO_3_ (Thermo Fisher Scientific, Illkirch, France), rinsed three times with Milli-Q water, filled with 0.1% HCl (TraceSelect, Fluka) and rinsed three times with seawater from the site. For each sampling, an aliquot of seawater was filtered in a 125 mL-FEP bottle, over a 0.2 *µ*m filter (pre-rinsed cellulose acetate syringe filter; Sartorius, Aubagne, France) in ordered to identify and quantify metals in the dissolved fraction, while a second aliquot was directly poured into a 125 mL-FEP bottle for the total acid leachable fraction latter considered as the total fraction. All samples were then acidified with 0.2% HNO_3_ (Suprapur; Merck) and UV-irradiated (150-W mercury lamp; Hanau, Germany) for at least 24h to degrade organic matter [1] and to assess the concentration of metals by stripping voltammetry.

For each sampling, subsamples (24 mL) for dissolved organic carbon (DOC) and total nitrogen (TN) analyses were collected from the seawater filtered over a 0.2 *µ*m filter (pre-rinsed cellulose acetate syringe filter; Sartorius, Aubagne, France). Subsamples were stored in pre-calcined (4h, 450°C) glass tubes with a Teflon/silicon septum (Wheaton), and preserved in 0.012M HCl (TraceSelect, Fluka) at 4°C until analysis.

## Supplementary information for dissolved organic carbon and total nitrogen analyses

Dissolved organic carbon (DOC) and total nitrogen (TN) analyses were performed using a TOC-VCSH analyzer. DOC and TN measurements are based on a high temperature catalytic oxidation followed by either an infrared (DOC) or a chemiluminescence detection (TN). The analyses were validated using certified reference materials (SUPER 05, Canada).

## Supplementary information for dissolved and total concentrations trace metals analyses

Dissolved and total concentrations of trace metals were obtained by differential pulse anodic stripping voltammetry (DPASV). All measurements were performed using an Autolab III potentiostat (Metrohm, Courtaboeuf, France) equipped with an autosampler.

## Supplementary information for flow cytometry analysis

Epiphytic cells were collected from algal samples by gently scraping the surface with sterile scalpels. Replicates of algal samples (*n* = 3) were fixed in 4 mL of 0.25% glutaraldehyde-sterile filtered seawater solution. Seawater samples (15 mL) were prefiltered using 40 *µ*m cell strainers and fixed with 0.25% of glutaraldehyde. All samples were kept at -80°C until analysis. For algal samples, epiphytic cells potentially aggregated together were dissociated using a sonication step of 2 min. Heterotrophic prokaryotes were stained using SYBR green I (Invitrogen, Carlsbad, CA, USA) and enumerated using a BD Accuri C6 flow cytometer (BD Biosciences, San Jose, CA, USA) as previously described [2]. Heterotrophic prokaryotes were discriminated as described in Fig. S1. Results were expressed as densities of cells per cm^2^ using the measured surface of each thallus estimated with the Mesurim pro software (v. 3.4).

## Supplementary information for 16S rRNA gene metabarcoding analyses (FROGS pipeline)

Each PCR reaction (25 *µ*L) was conducted using 12.5 *µ*L of GoTaq® Long PCR Master Mix (2x buffer), 1 *µ*L of each primer (10 *µ*M), 2 *µ*L of DNA (~30 ng.*µ*L^-1^), and 8.5 *µ*L of Nuclease-Free Water. The following thermal cycling scheme was conducted as followed: initial denaturation at 95◦C for 5 min, 25 cycles of denaturation at 95◦C for 45s, annealing at 50°C for 1 min, followed by extension at 72°C for 1 min. The final extension was carried out at 72°C for 10 min. Negative controls were performed by using the PCR reaction mixture without DNA (Nuclease-Free Water instead). PCR products were checked on a 1% agarose gel, following a staining step with the GelRed® Nucleic Acid Stain. Amplicons were cleaned and concentrated using 1X magnetic Agencourt AMPure XP beads (Beckman Coulter, Brea, CA). Concentrated DNA was quantified by PicoGreen fuorescence assay (Quant-iT PicoGreen® dsDNA Assay Kit, ThermoFischer Scientifc) and pooled at equimolar concentrations.

Sequences were filtered by removing those for which primers sequences were not present. The primer search accepts 10% of differences. Primers sequences were then removed in the remaining sequence using “cutadapt”. Then, merged sequences with length below 300 pb and above 500 pb were removed. Clustering step was performed using SWARM with a clustering aggregation distance set to 3 [3]. Chimeric sequences were removed *de novo* using VSEARCH [4]. Rare OTUs representing less than 0.005% of all sequences were removed. OTUs were affiliated with the silva132 16S rRNA gene database. The final matrix was obtained by removing all sequences affiliated to 16S rRNA gene from chloroplasts and mitochondria which represented an average of 37.5% (± 23.5%) of total sequences per samples (mostly chloroplasts). For *α*-diversity analyses only, the dataset was analyzed, by performing a rarefaction to the minimum library size which correspond to 5 827 sequences, using the “phyloseq” R package [5]. No rarefaction steps were conducted for compositional analyses, and the dataset was normalized to the total number of sequences per samples as recommended by [6, 7], since the removal of rare taxa counts was suggested to possibly impact the whole compositional analysis.

To investigate the effect of seawater abiotic parameters on the *β*-diversity of the algal epibacterial community, a db-RDA (Bray-Curtis distance) was performed using the rarefied OTU-table and the following seawater parameters: temperature, pH, salinity, [NO_3_^-^], [PO_4_^3-^], [Si(OH)_4_], DOC, TN, together with [Zn], [Pb], [Cd] and [Cu] (dissolved and total fractions). Oxygen measures were not used due to lacking values. As described in [8], significant constraints were selected using “ordiR2step” constraints (999 permutations). High redundancy was observed between dissolved and total concentrations of trace metals, and only the dissolved ones were kept for the final analysis. Finally, only temperature, [PO_4_^3-^], [NO_3_^-^], [Si(OH)_4_], DOC, TN, [Cu] and [Pb] were kept in the db-RDA final model. The overall significance of this model was then assessed using the *anova.cca()* function with 999 permutations using the “vegan” R package [9].

## Supplementary information for metabolomics analyses

The surface extracts were concentrated to dryness under reduced pressure at a temperature lower than 35°C [10]. Dried surface extracts were then transferred in 2-mL HPLC vials and stored under inert atmosphere (N_2_) in the dark at -20°C until analysis. Before injection, samples were solubilized in 1mL of LC-MS grade methanol (Chromasolv, Sigma-Aldrich, St Louis, MO, USA). For all experiments, extraction and sample preparation were carried out by the same operator. Analytical blanks were prepared with exactly the same protocol as those used for surface extracts but without algal pieces. These blanks allowed the subsequent subtraction of contaminants or components coming from solvents and vials. In order to ensure quality control, a pool sample was prepared by combining 100 *μ*L of each surface extract. The pool sample was divided into 18 2-ml HPLC vials (around 250 *µ*L of solution in each vial) that were used as quality-control samples (QCs). To ensure analytical repeatability, a first QC was injected ten times at the beginning of the sequence in order to stabilize the chromatographic system. Then, the injection sequence consisted of the iterative injection of one QC and five samples (surface extracts and analytical blanks) randomly selected to avoid any possible time-dependent changes in LC−MS chromatographic fingerprints. Moreover, to assess sample carry-over of the analytical process, two solvent blanks (LC-MS grade methanol) were injected before the first QC and at the end of the injection sequence.

The UPLC-HRMS instrumentation consisted of a Dionex Ultimate 3000 Rapid Separation (Thermo Fisher Scientific, Waltham, MA, USA) chromatographic system equipped with a RS pump, a temperature-controlled autosampler, a thermostated column compartment and an UV-Vis diode array detector. This system was coupled to a QToF Impact II mass spectrometer (Bruker Daltonics, Bremen, Germany). The analyses were performed using an analytical core-shell reversed-phase column (150 × 2.1 mm, 1.7 *μ*m, Kinetex Phenyl-Hexyl equipped with a SecurityGuard cartridge; Phenomenex, Le Pecq, France) with a column temperature of 40°C and a flow rate of 0.5 mL.min^-1^. The autosampler temperature was set at 4°C and the injection volume was 5 *μ*L. Mobile phases were: (A) water and (B) acetonitrile (Chromasolv; Sigma-Aldrich-Merck, Darmstadt, Germany) containing each 0.1% (*v*/*v*) of formic acid (Ultra grade; Fluka, Fischer Scientific, Illkirch, France). The elution gradient started at 5% B and kept for 2 min, then to 100% B (linear ramp) in 8 min and kept for 4 min; then back to 5% B (linear ramp) over 0.01 min and maintained 1.99 min, for a total run time of 16 min.

The capillary voltage of the MS spectrometer was set at 4500V (positive mode), and the nebulizing parameters were set as follows: nebulizing gas (N_2_) pressure at 0.4 bar, drying gas (N_2_) flow at 4 L.min^-1^, and drying temperature at 180°C. Mass spectra were recorded from *m/z* 50 to 1200 at a mass resolving power of 25 000 full width at half-maximum (FWHM, *m/z* = 200) and a frequency of 2 Hz. Tandem mass spectrometry analyses were performed thanks to a collision induced dissociation (CID) with a collision energy of 25 eV. A solution of formate/acetate forming clusters was automatically injected before each sample for internal mass calibration, and the mass spectrometer was calibrated with the same solution before each sequence of samples. Data handling was done using DataAnalysis software (version 4.3, Bruker Daltonics).

LC−MS raw data were automatically recalibrated using the calibration clusters found at the beginning of each chromatogram and converted into netCDF files (centroid mode) with a script developed within the DataAnalysis software. Converted files were preprocessed with the XCMS package (version 1.46.0) [11] in the R 3.2.3 environment: i) Peak picking was performed with the “centwave” method (peakwidth = c(2,20), ppm = 2) without threshold prefilter [12], ii) retention time correction was done with the “obiwarp” method (profstep = 0.1), iii) peaks were grouped using the following parameters bw = 10, minfrac = 0.5 and minsamp = 1, and iv) missing peaks were filled with default parameters.

To ensure data quality and remove redundant signals, three successive filtering steps were applied to preprocessed data using a R script described in [13]. The first was based on the signal/noise (S/N) ratio to remove signals of QCs observed in analytical blanks (ratio set at 10 for features matching between QCs and analytical blanks). The second allowed suppression of signals based on the value of the coefficient of variation (CV) of the intensity of the variables in the QCs (cutoff set at 20%). A third filtering step was applied using the coefficient of the autocorrelation (with a cutoff set at 80%) between variables with a same retention time in the surface extract samples.

## Supplementary information for the annotation procedure

In this study, three levels of annotation were applied for the identification of metabolites, following the same methodology described in [14, 15].

The first approach, which corresponds to the level 1 of annotation according to [16], was based on the use of several commercial standards described in [17] including dimethylsulfoniopropionate (DMSP), proline betaine, proline and mannitol, as well as compounds previously purified from *T. atomaria* or *Dictyota* spp. by our team [18, 19] including geranylgeranylglycerol (GGG), several sesquiterpenes and fucoxanthin. These standards were solubilized in MeOH at a concentration of 0.1 mg.mL^-1^ and analyzed with the same experimental conditions used for the three metabolomics workflows. All commercial standards were purchased from Sigma-Aldrich or Cayman Chemicals.

The second strategy was to annotate putatively some *m/z* features by the comparison of their MS (and MS/MS for LC-MS analyses) data with reference databases. A match found with a MS (and MS/MS) library is considered with the level 2 of annotation [16]. To facilitate the dereplication procedure, the molecular networking approach was used and allowed the clustering of metabolites with a similar chemical structure in a same sub-network (cluster) [20]. Briefly, molecular networks were generated using GNPS platform (<https://gnps.ucsd.edu/ProteoSAFe/static/gnps-splash.jsp>) [20]. MS/MS raw data (.mzxml files) were clustered using MS cluster with a tolerance of 0.02 Da for precursor ions and 0.02 Da for MS/MS fragment ions. Minimum cosine score (CS) value used for the clustering was set to 0.7. Data were exported and analyzed using Cytoscape (version 3.4.0). The resulting molecular network was analyzed and annotated based on MS/MS fragmentation pathways and comparison with in-house and public databases such as Metlin (https://metlin.scripps.edu/) or Lipidmaps (https://www.lipidmaps.org/).

When no characteristic fragmentation pathway was determined by comparison to the literature, the most probable chemical formula was proposed using the “Smart Formula” tool from DataAnalysis. This last step finally corresponded to the level 4 of annotation [16].

## Supplementary information for multi-omics analysis

The MixOmics R package [21] was used to perform the integration of environmental parameters together with 16S rRNA gene metabarcoding, and surface metabolomics datasets. For the metabolomics dataset, the matrix used was log_10_-transformed and mean-centered. For the 16S rRNA gene metabarcoding dataset, the matrix used corresponded to the final OTU-table normalized to the total number of sequences per samples, obtained without rarefaction step. For the environmental dataset, parameters kept in the data matrix were those selected in the final model db-RDA model as described in the supplementary section for 16S rRNA gene metabarcoding analysis.

The whole analysis was performed according to the DIABLO framework which is fully described with a case study here: <http://mixomics.org/mixdiablo/case-study-tcga/>. Briefly, the aim of this approach is to perform an optimal N-integration with a sparse method developed to reveal correlation between variables from heterogeneous datasets. The sparse PLS discriminant analysis allowed here to performs variable selection and classification in a one-step procedure with a PLS regression, and was used specifically here since each “omic” analysis performed corresponded to large data sets where Linear Discriminant Analysis faces collinearity issues. The analysis was performed by using a design matrix where all blocks were connected by the same link (0.1). The tuning to select the optimal number of components for each dataset was performed using the *perf()* function and resulted in 8 components for the metabarcoding and metabarcoding datasets, respectively, and 6 components for the environmental datasets. Then, the optimal number of variables per dataset was selected using the *tune.block.splsda()* function, allowing to obtain a sufficient number of variables for downstream validation/interpretation. The final model was then performed with the *block.splsda()* function. The *plotDiablo()* function allowed to determined correlations between each dataset. From this model, correlations were above 0.7 between all datasets. To visualize correlation between each variable of each dataset, the *network()* function was used with a correlation cutoff threshold set at 0.7. Subsequently, a total of 32 metabolites, 37 OTUs, and 2 environmental parameters were selected for the network structuration with only positive correlations above 0.7 and negative correlations below -0.7. The resulting network was exported using “igraph” R package and analyzed using Cytoscape.

## References for supplementary information

1. Omanović D, Kwokal Ž, Goodwin A, Lawrence A, Banks CE, Compton RG, et al. Trace metal detection in Šibenik Bay, Croatia: Cadmium, lead and copper with anodic stripping voltammetry and manganese via sonoelectrochemistry. A case study. *J Iran Chem Soc* 2006; **3**: 128–139.

2. Pollet T, Berdjeb L, Garnier C, Durrieu G, Le Poupon C, Misson B, et al. Prokaryotic community successions and interactions in marine biofilms: the key role of Flavobacteriia. *FEMS Microbiol Ecol* 2018; **94**.

3. Mahé F, Rognes T, Quince C, Vargas C de, Dunthorn M. Swarm: robust and fast clustering method for amplicon-based studies. *PeerJ* 2014; **2**: e593.

4. Rognes T, Flouri T, Nichols B, Quince C, Mahé F. VSEARCH: a versatile open source tool for metagenomics. *PeerJ* 2016; **4**: e2584.

5. McMurdie PJ, Holmes S. phyloseq: an R package for reproducible interactive analysis and graphics of microbiome census data. *PLoS One* 2013; **8**: e61217.

6. McMurdie PJ, Holmes S. Waste not, want not: why rarefying microbiome data is inadmissible. *PLoS Comput Biol* 2014; **10**: e1003531.

7. Gloor GB, Macklaim JM, Pawlowsky-Glahn V, Egozcue JJ. Microbiome datasets are compositional: and this is not optional. *Front Microbiol* 2017; **8**: 2224.

8. Coclet C, Garnier C, Durrieu G, Omanović D, D’Onofrio S, Le Poupon C, et al. Changes in bacterioplankton communities resulting from direct and indirect interactions with trace metal gradients in an urbanized marine coastal area. *Front Microbiol* 2019; **10**: 257.

9. Oksanen J, Blanchet FG, Friendly M, Kindt R, Legendre P, McGlinn D, et al. vegan: Community ecology package. 2019.

10. Othmani A, Briand J-F, Ayé M, Molmeret M, Culioli G. Surface metabolites of the brown alga *Taonia atomaria* have the ability to regulate epibiosis. *Biofouling* 2016; **32**: 801–813.

11. Smith CA, Want EJ, O’Maille G, Abagyan R, Siuzdak G. XCMS: processing mass spectrometry data for metabolite profiling using nonlinear peak alignment, matching, and identification. *Anal Chem* 2006; **78**: 779–787.

12. Patti GJ, Tautenhahn R, Siuzdak G. Meta-analysis of untargeted metabolomic data from multiple profiling experiments. *Nat Protoc* 2012; **7**: 508–516.

13. Favre L, Ortalo-Magné A, Greff S, Pérez T, Thomas OP, Martin J-C, et al. Discrimination of four marine biofilm-forming bacteria by LC-MS metabolomics and influence of culture parameters. *J Proteome Res* 2017; **16**: 1962–1975.

14. Paix B, Othmani A, Debroas D, Culioli G, Briand J-F. Temporal covariation of epibacterial community and surface metabolome in the Mediterranean seaweed holobiont *Taonia atomaria*. *Environ Microbiol* 2019; **21**: 3346–3363.

15. Paix B, Carriot N, Barry-Martinet R, Greff S, Misson B, Briand J-F, et al. A multi-omics analysis suggests links between the differentiated surface metabolome and epiphytic microbiota along the thallus of a Mediterranean seaweed holobiont. *Front Microbiol* 2020; **11**: 494.

16. Schymanski EL, Jeon J, Gulde R, Fenner K, Ruff M, Singer HP, et al. Identifying small molecules via high resolution mass spectrometry: Communicating confidence. *Environ Sci Technol* 2014; **48**: 2097–2098.

17. Paix B, Ezzedine JA, Jacquet S. Diversity, dynamics, and distribution of *Bdellovibrio* and like organisms in perialpine lakes. *Appl Environ Microbiol* 2019; **85**: e02494-18.

18. Othmani A, Bunet R, Bonnefont J-L, Briand J-F, Culioli G. Settlement inhibition of marine biofilm bacteria and barnacle larvae by compounds isolated from the Mediterranean brown alga *Taonia atomaria*. *J Appl Phycol* 2016; **28**: 1975–1986.

19. Viano Y, Bonhomme D, Camps M, Briand J-F, Ortalo-Magné A, Blache Y, et al. Diterpenoids from the Mediterranean brown alga *Dictyota* sp. evaluated as antifouling substances against a marine bacterial biofilm. *J Nat Prod* 2009; **72**: 1299–1304.

20. Wang M, Carver JJ, Phelan VV, Sanchez LM, Garg N, Peng Y, et al. Sharing and community curation of mass spectrometry data with Global Natural Products Social Molecular Networking. *Nat Biotechnol* 2016; **34**: 828–837.

21. Lê Cao K-A, González I, Déjean S. integrOmics: an R package to unravel relationships between two omics datasets. *Bioinformatics* 2009; **25**: 2855–2856.

# Supplementary Tables

## Table S1. Environmental parameters and sampling dates (Part 1: from February to April 2017). For trace metals, (d) and (t) indicated dissolved and total acid leachable fractions, respectively. n.d.: not determined.

| **Month** | **Site** | **Date** | **Temperature** | **O_2_** | **pH** | **Salinity** | **N-NO_3_^-^** | **SI(OH)_4_** | **PO_4_^3-^** | **DOC** | **TN** | **Zn (d)** | **Zn (t)** | **Pb (d)** | **Pb (t)** | **Cd (d)** | **Cd (t)** | **Cu (d)** | **Cu (t)** |
| --- | --- | --- | --- | --- | --- | --- | --- | --- | --- | --- | --- | --- | --- | --- | --- | --- | --- | --- | --- |
|  |  | (dd/mm) | (°C) | (%) | - | (ppt) | (*µ*M) | (*µ*M) | (*µ*M) | (mg C.L^-1^) | (mg N.L^-1^) | (nM) | (nM) | (nM) | (nM) | (nM) | (nM) | (nM) | (nM) |
| M1  Feb. | S1 | 15/02 | 12.9 | n.d. | 8.2 | 37.8 | 0.38 ± 0.05 | 0.88 ± 0.04 | 0.33 ± 0.06 | 1.03 ± 0.01 | 0.10 ± 0.001 | 61.0 | 64.4 | 1.65 | 4.35 | 0.13 | 0.14 | 22.1 | 23.1 |
|  | S2 | 15/02 | 13.8 | n.d. | 8.2 | 38.1 | 0.38 ± 0.05 | 0.63 ± 0.03 | 0.23 ± 0.01 | 0.98 ± 0.01 | 0.07 ± 0.003 | 7.6 | 6.7 | 0.12 | 0.28 | 0.06 | 0.05 | 3.3 | 4.1 |
|  | S3 | 21/02 | 12.3 | n.d. | 8.4 | 38.6 | 0.51 ± 0.06 | 0.28 ± 0.04 | 0.27 ± 0.04 | 0.99 ± 0.01 | 0.07 ± 0.002 | 6.9 | 7.5 | 0.14 | 0.12 | 0.05 | 0.06 | 3.8 | 3.7 |
|  | S4 | 21/02 | 11.5 | n.d. | 8.4 | 38.6 | 0.51 ± 0.05 | 0.28 ± 0.04 | 0.26 ± 0.01 | 1.01 ± 0.02 | 0.09 ± 0.008 | 5.1 | 4.3 | 0.08 | 0.09 | 0.05 | 0.06 | 2.7 | 2.9 |
|  | S5 | 23/02 | 14.7 | n.d. | 8.2 | 38.4 | 0.38 ± 0.05 | 0.48 ± 0.07 | 0.22 ± 0.03 | 1.13 ± 0.01 | 0.09 ± 0.004 | 50.3 | 53.1 | 1.02 | 1.48 | 0.12 | 0.12 | 4.4 | 4.8 |
| M2  Mar. | S1 | 27/03 | 15.8 | 103 | 7.8 | 36.5 | 0.44 ± 0.05 | 0.46 ± 0.04 | 0.23 ± 0.06 | 1.11 ± 0.01 | 0.11 ± 0.002 | 48.0 | 65.9 | 1.29 | 5.20 | 0.11 | 0.12 | 20.8 | 28.1 |
|  | S2 | 27/03 | 14.9 | 84 | 7.9 | 37.2 | 0.38 ± 0.05 | 0.59 ± 0.03 | 0.19 ± 0.07 | 1.03 ± 0.01 | 0.07 ± 0.002 | 7.4 | 7.9 | 0.14 | 0.34 | 0.07 | 0.04 | 3.7 | 4.3 |
|  | S3 | 23/03 | 15.5 | 83 | 8.3 | 38.3 | 0.35 ± 0.05 | 0.15 ± 0.16 | 0.18 ± 0.03 | 1.00 ± 0.01 | 0.07 ± 0.004 | 9.0 | 10.7 | 0.12 | 0.19 | 0.06 | 0.07 | 3.8 | 4.5 |
|  | S4 | 23/03 | 14.3 | 84 | 7.9 | 38.2 | 0.38 ± 0.05 | 0.40 ± 0.10 | 0.19 ± 0.05 | 1.02 ± 0.01 | 0.08 ± 0.004 | 6.8 | 7.5 | 0.09 | 0.25 | 0.07 | 0.07 | 3.1 | 3.7 |
|  | S5 | 27/03 | 14.4 | 94 | 8.0 | 37.1 | 0.35 ± 0.05 | 0.57 ± 0.05 | 0.18 ± 0.03 | 1.12 ± 0.01 | 0.18 ± 0.002 | 109.3 | 149.4 | 1.81 | 4.28 | 0.16 | 0.24 | 4.7 | 6.9 |
| M3  Apr. | S1 | 18/04 | 16.9 | 119 | 8.1 | 36.8 | 0.35 ± 0.04 | 0.52 ± 0.09 | 0.34 ± 0.04 | 1.10 ± 0.02 | 0.09 ± 0.002 | 32.4 | 39.2 | 1.18 | 3.04 | 0.08 | 0.09 | 14.8 | 19.0 |
|  | S2 | 18/04 | 16.0 | 95 | 8.2 | 37.0 | 0.32 ± 0.04 | 0.57 ± 0.02 | 0.18 ± 0.01 | 1.07 ± 0.01 | 0.08 ± 0.002 | 6.1 | 8.2 | 0.14 | 0.44 | 0.05 | 0.05 | 4.1 | 5.4 |
|  | S3 | 27/04 | 14.6 | 88 | 7.7 | 37.2 | 0.28 ± 0.04 | 0.87 ± 0.01 | 0.36 ± 0.11 | 1.10 ± 0.01 | 0.26 ± 0.004 | 10.0 | 9.4 | 0.12 | 0.23 | 0.06 | 0.06 | 5.2 | 6.0 |
|  | S4 | 27/04 | 14.4 | 84 | 7.8 | 37.2 | 0.22 ± 0.04 | 0.22 ± 0.12 | 0.26 ± 0.06 | 1.07 ± 0.01 | 0.08 ± 0.001 | 10.6 | 10.4 | 0.07 | 0.23 | 0.07 | 0.06 | 2.8 | 3.7 |
|  | S5 | 18/04 | 15.2 | 86 | 8.0 | 37.3 | 0.28 ± 0.04 | 0.21 ± 0.01 | 0.28 ± 0.08 | 1.16 ± 0.01 | 0.10 ± 0.004 | 338.9 | 348.3 | 3.65 | 7.46 | 0.43 | 0.50 | 6.7 | 8.7 |

| **Month** | **Site** | **Date** | **Temperature** | **O_2_** | **pH** | **Salinity** | **N-NO_3_^-^** | **SI(OH)_4_** | **PO_4_^3-^** | **DOC** | **TN** | **Zn (d)** | **Zn (t)** | **Pb (d)** | **Pb (t)** | **Cd (d)** | **Cd (t)** | **Cu (d)** | **Cu (t)** |
| --- | --- | --- | --- | --- | --- | --- | --- | --- | --- | --- | --- | --- | --- | --- | --- | --- | --- | --- | --- |
|  |  | (dd/mm) | (°C) | (%) | - | (ppt) | (*µ*M) | (*µ*M) | (*µ*M) | (mg C.L^-1^) | (mg N.L^-1^) | (nM) | (nM) | (nM) | (nM) | (nM) | (nM) | (nM) | (nM) |
| M4  May | S1 | 29/05 | 22.1 | 90 | 8.1 | 36.6 | 0.28 ± 0.04 | 0.58 ± 0.09 | 0.18 ± 0.05 | 1.32 ± 0.01 | 0.09 ± 0.003 | 45.3 | 109.2 | 1.50 | 15.21 | 0.08 | 0.12 | 37.5 | 63.2 |
|  | S2 | 29/05 | 21.6 | 88 | 7.9 | 36.8 | 0.28 ± 0.04 | 0.34 (SD n.d.) | 0.12 ± 0.02 | 1.34 ± 0.01 | 0.16 ± 0.001 | 14.0 | 15.9 | 0.74 | 0.28 | 0.06 | 0.06 | 6.2 | 6.5 |
|  | S3 | 18/05 | 18.4 | 93 | 8.2 | 36.8 | 0.25 ± 0.04 | 0.64 ± 0.21 | 0.10 ± 0.02 | 1.07 ± 0.01 | 0.08 ± 0.003 | 10.0 | 10.8 | 0.11 | 0.17 | 0.06 | 0.06 | 3.2 | 3.4 |
|  | S4 | 18/05 | 17.1 | 92 | 8.2 | 36.9 | 0.28 ± 0.04 | 0.40 ± 0.12 | 0.09 ± 0.01 | 1.10 ± 0.02 | 0.07 ± 0.001 | 7.1 | 5.8 | 0.10 | 0.24 | 0.06 | 0.06 | 2.7 | 3.0 |
|  | S5 | 29/05 | 19.9 | 85 | 8.0 | 36.8 | 0.28 ± 0.04 | 0.27 ± 0.08 | 0.06 ± 0.01 | 1.12 ± 0.01 | 0.08 ± 0.002 | 78.2 | 92.9 | 1.74 | 2.70 | 0.18 | 0.20 | 4.4 | 5.3 |
| M5  Jun. | S1 | 27/06 | 24.3 | 82 | 8.0 | 36.9 | 0.29 ± 0.02 | 0.75 ± 0.02 | 0.10 ± 0.02 | 1.04 ± 0.01 | 0.11 ± 0.010 | 27.6 | 56.0 | 1.14 | 8.51 | 0.08 | 0.10 | 20.9 | 39.1 |
|  | S2 | 27/06 | 23.2 | 88 | 8.0 | 36.8 | 0.38 ± 0.02 | 0.75 ± 0.03 | 0.06 ± 0.01 | 1.06 ± 0.01 | 0.12 ± 0.010 | 8.8 | 11.0 | 0.17 | 0.41 | 0.06 | 0.06 | 6.1 | 6.5 |
|  | S3 | 21/06 | 23.6 | 94 | 7.9 | 36.9 | 0.35 ± 0.02 | 0.72 ± 0.07 | 0.16 ± 0.06 | 0.98 ± 0.02 | 0.08 ± 0.010 | 13.1 | 12.0 | 0.25 | 0.15 | 0.06 | 0.07 | 7.6 | 6.6 |
|  | S4 | 21/06 | 23.8 | 84 | 7.9 | 36.9 | 0.32 ± 0.02 | 0.5 ± 0.04 | 0.12 ± 0.06 | 1.16 ± 0.02 | 0.11 ± 0.010 | 7.5 | 7.9 | 0.10 | 0.17 | 0.08 | 0.10 | 4.0 | 3.8 |
| M6  Jul. | S2 | 18/07 | 23.1 | 89 | 8.0 | 37.1 | 0.63 ± 0.05 | 0.58 ± 0.04 | 0.23 ± 0.02 | 1.15 ± 0.01 | 0.10 ± 0.001 | 6.8 | 15.4 | 0.11 | 0.18 | 0.07 | 0.06 | 6.3 | 6.4 |
|  | S4 | 12/07 | 21.3 | 80 | 8.0 | 37.0 | 0.65 ± 0.05 | 0.36 ± 0.02 | 0.26 ± 0.01 | 1.11 ± 0.02 | 0.08 ± 0.001 | 10.6 | 8.8 | 0.05 | 0.11 | 0.06 | 0.07 | 3.3 | 3.4 |

## Table S1. Environmental parameters and sampling dates (Part 2 from May to July 2017). For trace metals, (d) and (t) indicated dissolved and total acid leachable fractions, respectively. n.d.: not determined.

## Table S2. Multivariate pairwise results (*p* values) examining differences between “sample types” for the 16S rRNA gene dataset with all samples (999 permutations).

|  | **Rocky biofilms** | ***T. atomaria*** |
| --- | --- | --- |
| ***T. atomaria*** | 0. | **-** |
| **Seawater** | 0.001 | 0.001 |

## Table S3. Multivariate pairwise results (*p* values) examining differences between “months” and between “sites” for the 16S rRNA gene dataset with *Taonia atomaria* samples (999 permutations).

| **Comparison between months** | | | | | |  | **Comparison between sites** | | | | |
| --- | --- | --- | --- | --- | --- | --- | --- | --- | --- | --- | --- |
|  | **M1** | **M2** | **M3** | **M4** | **M5** |  |  | **S1** | **S2** | **S3** | **S4** |
| **M2** | 0.001 | - | - | - | - |  | **S2** | 0.005 | - | - | - |
| **M3** | 0.001 | 0.003 | - | - | - |  | **S3** | 0.003 | 0.026 | - | - |
| **M4** | 0.001 | 0.001 | 0.002 | - | - |  | **S4** | 0.003 | 0.010 | 0.084 | - |
| **M5** | 0.001 | 0.001 | 0.001 | 0.001 | - |  | **S5** | 0.003 | 0.016 | 0.020 | 0.016 |
| **M6** | 0.001 | 0.001 | 0.001 | 0.002 | 0.042 |  |  |  |  |  |  |

## Table S4. Multivariate pairwise results (*p* values) examining differences between “months” and between “sites” for the 16S rRNA gene dataset with rocky biofilm samples (999 permutations).

| **Comparison between months** | | | | | |  | **Comparison between sites** | | | | |
| --- | --- | --- | --- | --- | --- | --- | --- | --- | --- | --- | --- |
|  | **M1** | **M2** | **M3** | **M4** | **M5** |  |  | **S1** | **S2** | **S3** | **S4** |
| **M2** | 0.930 | - | - | - | - |  | **S2** | 0.070 | - | - | - |
| **M3** | 0.140 | 0.270 | - | - | - |  | **S3** | 0.070 | 0.070 | - | - |
| **M4** | 0.140 | 0.140 | 0.570 | - | - |  | **S4** | 0.070 | 0.250 | 0.930 | - |
| **M5** | 0.140 | 0.270 | 0.270 | 0.640 | - |  | **S5** | 0.070 | 0.200 | 0.260 | 0.600 |
| **M6** | 0.270 | 0.290 | 0.640 | 0.640 | 0.640 |  |  |  |  |  |  |

## Table S5. Multivariate pairwise results (*p* values) examining differences between “months” and between “sites” for the 16S rRNA gene dataset with seawater samples (999 permutations).

| **Comparison between months** | | | | | |  | **Comparison between sites** | | | | |
| --- | --- | --- | --- | --- | --- | --- | --- | --- | --- | --- | --- |
|  | **M1** | **M2** | **M3** | **M4** | **M5** |  |  | **S1** | **S2** | **S3** | **S4** |
| **M2** | 0.232 | - | - | - | - |  | **S2** | 0.300 | - | - | - |
| **M3** | 0.041 | 0.041 | - | - | - |  | **S3** | 0.280 | 0.880 | - | - |
| **M4** | 0.041 | 0.041 | 0.048 | - | - |  | **S4** | 0.280 | 0.370 | 0.390 | - |
| **M5** | 0.041 | 0.041 | 0.031 | 0.041 | - |  | **S5** | 0.230 | 0.750 | 0.910 | 0.230 |
| **M6** | 0.061 | 0.070 | 0.070 | 0.135 | 1.000 |  |  |  |  |  |  |

## Table S6. SIMPER results of the most contributing genera to the dissimilarities between seawater and algal samples. Only the first 50% of the cumulative contribution is shown. *p* values were calculated with a permutation test constructed with 999 permutations and corresponded to the probability of getting a larger or equal average contribution in random permutation of the group factor. Cum. sum corresponded to the ordered cumulative contribution.

| **Order** | **Family** | **Genus** | **Average contribution** | **SD** | **Av. water samples** | **Av. algal samples** | **Cum. sum** | ***p* value** |
| --- | --- | --- | --- | --- | --- | --- | --- | --- |
| *Synechococcales* | *Cyanobiaceae* | *Synechococcus* | 6.8% | 0.06 | 13.6% | 0.0% | 8.3% | 0.001 |
| *Caulobacterales* | *Hyphomonadaceae* | *Litorimonas* | 3.5% | 0.04 | 0.2% | 7.2% | 12.6% | 0.256 |
| *Rhodobacterales* | *Rhodobacteraceae* | *Litorimicrobium* | 2.9% | 0.04 | 5.7% | 0.0% | 20.0% | 0.001 |
| *Thiohalorhabdales* | *Thiohalorhabdaceae* | *Granulosicoccus* | 2.8% | 0.03 | 0.5% | 6.1% | 23.5% | 0.081 |
| *Rhizobiales* | *Rhizobiaceae* | *Nitratireductor* | 2.5% | 0.04 | 0.3% | 5.3% | 26.6% | 0.61 |
| *Chitinophagales* | *Saprospiraceae* | Unknown | 2.5% | 0.01 | 1.2% | 6.1% | 29.6% | 0.001 |
| *Flavobacteriales* | *Flavobacteriaceae* | *Algitalea* | 2.3% | 0.03 | 0.0% | 4.7% | 32.4% | 0.435 |
| *Pirellulales* | *Pirellulaceae* | Unknown | 2.3% | 0.02 | 4.6% | 0.0% | 35.2% | 0.001 |
| *Flavobacteriales* | *Flavobacteriaceae* | NS4 marine group | 2.2% | 0.04 | 4.4% | 0.0% | 37.9% | 0.001 |
| *Flavobacteriales* | *Flavobacteriaceae* | NS5 marine group | 2.0% | 0.04 | 3.9% | 0.0% | 40.3% | 0.001 |
| *Rhodobacterales* | *Rhodobacteraceae* | *Planktotalea* | 1.4% | 0.03 | 3.1% | 0.3% | 42.0% | 0.002 |
| *Verrucomicrobiales* | *Rubritaleaceae* | *Rubritalea* | 1.3% | 0.01 | 3.1% | 2.7% | 43.6% | 0.15 |
| *Flavobacteriales* | *Flavobacteriaceae* | *Formosa* | 1.3% | 0.02 | 2.7% | 0.0% | 45.3% | 0.001 |
| *Rhodobacterales* | *Rhodobacteraceae* | Unknown | 1.2% | 0.01 | 1.5% | 3.4% | 46.8% | 0.833 |
| *Flavobacteriales* | *Flavobacteriaceae* | *Croceitalea* | 1.1% | 0.01 | 0.5% | 2.6% | 48.1% | 0.049 |
| SAR11 clade | Clade I | Clade Ia | 1.1% | 0.03 | 2.2% | 0.0% | 49.4% | 0.001 |

## Table S7. SIMPER results of the most contributing genera to the dissimilarities between rocky biofilm samples and algal samples. Only the first 50% of the cumulative contribution is showed. *p* values were calculated with a permutation test constructed with 999 permutations and corresponded to the probability of getting a larger or equal average contribution in random permutation of the group factor. Cum. sum corresponded to the ordered cumulative contribution.

| **Order** | **Family** | **Genus** | **Average contribution** | **SD** | **Av. rocky biofilms** | **Av. algal samples** | **Cum. sum** | ***p* value** |
| --- | --- | --- | --- | --- | --- | --- | --- | --- |
| *Rhizobiales* | *Rhizobiaceae* | *Nitratireductor* | 2.6% | 0.04 | 0.1% | 5.3% | 13.2% | 0.545 |
| *Thiohalorhabdales* | *Thiohalorhabdaceae* | *Granulosicoccus* | 2.6% | 0.03 | 1.2% | 6.1% | 16.9% | 0.334 |
| *Flavobacteriales* | *Flavobacteriaceae* | *Algitalea* | 2.3% | 0.03 | 0.8% | 4.7% | 20.3% | 0.564 |
| *Rhodobacterales* | *Rhodobacteraceae* | *Loktanella* | 1.9% | 0.02 | 4.6% | 1.2% | 23.0% | 0.001 |
| *Chitinophagales* | *Saprospiraceae* | Unknown | 1.5% | 0.01 | 4.7% | 6.1% | 25.3% | 0.918 |
| *Flavobacteriales* | *Flavobacteriaceae* | *Maritimimonas* | 1.4% | 0.01 | 2.8% | 0.1% | 27.3% | 0.001 |
| *Rhodobacterales* | *Rhodobacteraceae* | Unknown | 1.4% | 0.01 | 4.4% | 3.4% | 29.3% | 0.173 |
| *Flavobacteriales* | *Flavobacteriaceae* | *Aquimarina* | 1.2% | 0.02 | 2.5% | 1.0% | 31.1% | 0.002 |
| *Chitinophagales* | *Saprospiraceae* | *Lewinella* | 1.2% | 0.01 | 3.8% | 1.9% | 32.9% | 0.001 |
| *Verrucomicrobiales* | *Rubritaleaceae* | *Rubritalea* | 1.2% | 0.01 | 0.5% | 2.7% | 34.7% | 0.584 |
| *Flavobacteriales* | *Flavobacteriaceae* | *Croceitalea* | 1.1% | 0.01 | 0.9% | 2.6% | 36.3% | 0.072 |
| *Flavobacteriales* | *Flavobacteriaceae* | *Winogradskyella* | 0.9% | 0.01 | 2.4% | 1.1% | 39.1% | 0.001 |
| *Chitinophagales* | *Saprospiraceae* | *Rubidimonas* | 0.9% | 0.01 | 0.9% | 2.3% | 40.4% | 0.119 |
| *Flavobacteriales* | *Flavobacteriaceae* | *Maribacter* | 0.9% | 0.01 | 1.7% | 1.7% | 41.7% | 0.381 |
| *Thiotrichales* | *Thiotrichaceae* | *Leucothrix* | 0.8% | 0.01 | 0.1% | 1.7% | 43.0% | 0.087 |
| *Rhodobacterales* | *Rhodobacteraceae* | *Litoreibacter* | 0.8% | 0.01 | 2.4% | 1.0% | 44.2% | 0.001 |
| *Nostocales* | *Xenococcaceae* | *Pleurocapsa* | 0.8% | 0.01 | 1.6% | 0.1% | 45.3% | 0.001 |
| *Flavobacteriales* | *Flavobacteriaceae* | *Tenacibaculum* | 0.8% | 0.01 | 0.5% | 1.7% | 46.5% | 0.299 |
| *Microtrichales* | *Microtrichaceae* | Sva0996 marine group | 0.7% | 0.01 | 0.4% | 1.6% | 47.5% | 0.542 |
| *Chitinophagales* | Unknown | Unknown | 0.7% | 0.01 | 0.9% | 1.3% | 48.5% | 0.116 |
| *Thalassobaculales* | Unknown | Unknown | 0.7% | 0.01 | 1.3% | 0.0% | 49.5% | 0.001 |

## Table S8. Multivariate pairwise results (*p* values) examining differences between “months” and between “sites” for the LC-(+)-ESI-MS metabolomics dataset (999 permutations).

| **Comparison between months** | | | | | |  | **Comparison between sites** | | | | |
| --- | --- | --- | --- | --- | --- | --- | --- | --- | --- | --- | --- |
|  | **M1** | **M2** | **M3** | **M4** | **M5** |  |  | **S1** | **S2** | **S3** | **S4** |
| **M2** | 0.001 | - | - | - | - |  | **S2** | 0.009 | - | - | - |
| **M3** | 0.001 | 0.009 | - | - | - |  | **S3** | 0.003 | 0.009 | - | - |
| **M4** | 0.001 | 0.001 | 0.009 | - | - |  | **S4** | 0.003 | 0.053 | 0.005 | - |
| **M5** | 0.001 | 0.001 | 0.001 | 0.001 | - |  | **S5** | 0.003 | 0.011 | 0.017 | 0.008 |
| **M6** | 0.001 | 0.001 | 0.001 | 0.001 | 0.001 |  |  |  |  |  |  |

## Table S9. List of biomarkers (VIP score > 2) identified by LC-HRMS and involved in the discrimination between months within surface extracts of *T. atomaria* (Part 1). *P* values corresponded to results of one-way ANOVA tests using “Month” as factor. Color codes corresponded to mean normalized concentrations (see Part2: Table S12).

| ***m/z*** | **Rt (s)** | **VIP score** | **Molecular formula** | **Adduct** | **Mass error (ppm)** | **mσ ^a^** | **Putative identification ^b^** | **MS/MS fragment ions** | ***p* values** | **M1** | **M2** | **M3** | **M4** | **M5** | **M6** |
| --- | --- | --- | --- | --- | --- | --- | --- | --- | --- | --- | --- | --- | --- | --- | --- |
| 704.5471 | 669 | 5.6 | C_42_H_74_NO_7_ | [M+H]^+^ | -0.7 | 21.4 | DGTA (C32:4) (C18:4/C14:0) | 704.5473 [M+H]^+^ ; 494.3488 [C_28_H_48_NO_6_]^+^ ; 476.3351 [C_28_H_46_NO_5_]^+^ ; 446.3483 [C_24_H_48_NO_6_]^+^ ; 428.3367 [C_24_H_46_NO_5_]^+^ ; 236.1494 [C_10_H_22_NO_5_]^+^ | 3.08E-14 |  |  |  |  |  |  |
| 490.3743 | 519 | 5.4 | C_26_H_52_NO_7_ | [M+H]^+^ | -0.2 | 9.2 | *lyso*-DGCC (C16:0) | 490.3744 [M+H]^+^ ; 313.2748 [C_19_H_37_O_3_]^+^ ; 132.1019 [C_6_H_14_NO_2_]^+^ ; 104.1069 [C_5_H_14_NO]^+^ | 2.67E-18 |  |  |  |  |  |  |
| 706.5626 | 682 | 4.7 | C_42_H_76_NO_7_ | [M+H]^+^ | -0.7 | 10.5 | DGTA (C32:3) (C18:3/C14:0) | 706.5621 [M+H]^+^ ; 496.3635 [C_28_H_50_NO_6_]^+^ ; 446.3479 [C_24_H_48_NO_6_]^+^ ; 236.1492 [C_10_H_22_NO_5_]^+^ | 8.06E-14 |  |  |  |  |  |  |
| 832.6098 | 688 | 4.0 | C_52_H_82_NO_7_ | [M+H]^+^ | -4.2 | -32.2 | DGTA (C44:10) (C20:4/C22:6) | 832.6121 [M+H]^+^ ; 548.3946 [C_32_H_54_NO_6_]^+^ ; 548.3798 [C_32_H_52_NO_6_]^+^ ; 528.3683 [C_32_H_50_NO_5_]^+^ ; 522.3799 [C_30_H_52_NO_6_]^+^ ; 520.3658 [C_30_H_50_NO_6_]^+^ ; 236.194 [C_10_H_22_NO_5_]^+^ | 2.29E-20 |  |  |  |  |  |  |
| 462.8933 | 475 | 3.3 | C_16_H_14_Br_2_ClO_4_ | [M+H]^+^ | 1.4 | ? | Hemichrysophaentin D? | 462.8933 [C_16_H_14_Br_2_ClO_4_]^+^ [M+H]^+^; 426.9164 [C_16_H_13_Br_2_O_4_]^+^ [M+H-HCl]^+^; 347.9987 [C_16_H_13_BrO_4_]^+^; 269.0807 [C_16_H_13_O_4_]^+^; 238.9708 [C_10_H_8_BrO_2_]^+^; 200.9553 [C_7_H_6_BrO_2_]^+^; 161.0599 [C_10_H_9_O_2_]^+^; 147.0446 [C_9_H_7_O_2_]^+^ | 2.31E-08 |  |  |  |  |  |  |
| 542.3237 | 524 | 3.3 | C_28_H_49_NO_7_P | [M+H]^+^ | -0.2 | 12.7 | *lyso*-PC (C20:5) | 542.3242 [M+H]^+^ ; 441.2975 [C_21_H_46_O_7_P]^+^ ; 184.0729 [C_5_H_15_NO_4_P]^+^ ; 104.1069 [C_5_H_14_NO]^+^ , 86.0964 [C_5_H_12_N]^+^ | 1.08E-09 |  |  |  |  |  |  |

^a^ Constructor statistical match factor (comparison of theoretical and experimental isotopic patterns); ^b^ Abbreviations: DGTA: diacylglycerylhydroxymethyl-*N*,*N*,*N*-trimethyl-*β*-alanine, DGCC: monoacylglyceryl-3-O-carboxy-(hydroxymethyl)-choline, PC: diacylglycerophosphocholine.

## Table S9. List of biomarkers (VIP score > 2) identified by LC-HRMS and involved in the discrimination between months within surface extracts of *T. atomaria* (Part 2). *P* values corresponded to results of one-way ANOVA tests using “Month” as factor. Color codes corresponded to mean normalized concentrations.

| ***m/z*** | **Rt (s)** | **VIP score** | **Molecular formula** | **Adduct** | **Mass error (ppm)** | **mσ ^a^** | **Putative identification ^b^** | **MS/MS fragment ions ^c^** | ***p* values** | **M1** | **M2** | **M3** | **M4** | **M5** | **M6** |
| --- | --- | --- | --- | --- | --- | --- | --- | --- | --- | --- | --- | --- | --- | --- | --- |
| 718.5621 | 683 | 3.1 | C_43_H_76_NO_7_ | [M+H]^+^ | 0.5 | 50.3 | DGTA (C33:3) ? | n.f.^e^ | 2.10E-09 |  |  |  |  |  |  |
| 706.5628 | 691 | 2.9 | C_42_H_78_NO_7_ | [M+H]^+^ | -1.4 | 13.9 | DGTA (C32:2) (C18:2/C14:0) (C16:1/C16:1) | 706.5633 [M+H]^+^ ; 496.3638 [C_28_H_50_NO_6_]^+^ ; 446.3482 [C_24_H_48_NO_6_]^+^ ; 236.1493 [C_10_H_22_NO_5_]^+^ | 5.53E-14 |  |  |  |  |  |  |
| 135.0476 | 54 | 2.9 | C_5_H_11_O_2_S | [M+H]^+^ | -1 | 6.1 | DMSP ^d^ | 135.0474 [M+H]^+^ ; 73.0282 [C_3_H_5_O_2_]^+^; 63.0261 [C_2_H_7_S]^+^ ; 61.0102 [C_2_H_5_S]^+^; 55.0177 [C_3_H_3_O]^+^ | 3.33E-06 |  |  |  |  |  |  |
| 546.3790 | 504 | 2.8 | C_32_H_52_NO_6_ | [M+H]^+^ | -1.5 | -57.4 | *lyso*-DGTA (C22:6) | 546.3797 [M+H]^+^ ; 236.1492 [C_10_H_22_NO_5_]^+^ | 2.71E-11 |  |  |  |  |  |  |
| 536.3574 | 504 | 2.8 | C_30_H_46_NO_7_ | [M+H]^+^ | 0.5 | 14.9 | C_30_H_45_NO_7_ | n.f. | 8.75E-14 |  |  |  |  |  |  |
| 696.5731 | 703 | 2.7 | C_41_H_78_NO_7_ | [M+H]^+^ | 0.3 | 31 | DGTA (C31:1) ? | n.f. | 1.51E-07 |  |  |  |  |  |  |
| 417.3365 | 708 | 2.6 | C_27_H_45_O_3_ | [M+H]^+^ | -0.4 | 287.3 | Tocopherol derivative ? | 207.1014 [C_12_H_15_O_3_]^+^; 193.0855 [C_11_H_13_O_3_]^+^; 153.0542 [C_8_H_9_O_3_]^+^; 137.0599 [C_8_H_9_O_2_]^+^ | 5.19E-08 |  |  |  |  |  |  |
| 565.4034 | 696 | 2.4 | C_40_H_53_O_2_ | [M+H]^+^ | 1.1 | 44.5 | Carotenoid ? | n.f. | 2.72E-09 |  |  |  |  |  |  |
| 219.1128 | 248 | 2.1 | C_12_H_15_N_2_O_2_ | [M+NH_4_]^+^ | 18.3 | 8.5 | C_12_H_15_N_2_O_2_ | 219.1127 [M+NH_4_]^+^; 217.1030 [C_12_H_13_N_2_O_2_]^+^; 202.0864 [C_12_H_12_NO_2_]^+^; 184.0753 [C_12_H_10_NO]^+^; 174.0969 [C_11_H_12_NO]^+^; 156.0805 [C_11_H_10_N]^+^; 146.0600 [C_9_H_8_NO]^+^; 132.0802 [C_9_H_10_N]^+^ | 4.59E-06 |  |  |  |  |  |  |
|  |  |  |  |  |  |  |  |  |  |  |  |  |  |  |  |
|  |  |  |  |  |  |  |  |  |  | Color code | | | | | |
|  |  |  |  |  |  |  |  |  |  | -3 | -1 | 1 | 3 | 5 | 7 |

^a^ Constructor statistical match factor (comparison of theoretical and experimental isotopic patterns); ^b^ Abbreviations: DGTA: diacylglycerylhydroxymethyl-*N*,*N*,*N*-trimethyl-*β*-alanine; DMSP: Dimethylsulfoniopropionate. ^c^ not observed; ^d^ This identification was confirmed with a commercial standard; ^e^ Not fragmented.

## Table S10. List of biomarkers (VIP score > 2) identified by LC-HRMS and involved in the discrimination between sites within surface extracts of *T. atomaria*. *P* values corresponded to results of one-way ANOVA tests using “Sites” as factor. Color codes corresponded to mean normalized concentrations.

| ***m/z*** | **Rt (s)** | **VIP score** | **Molecular formula** | **Adduct** | **Mass error (ppm)** | **mσ^a^** | **Putative identification** | **MS/MS fragment ions** | ***p* values** | **S1** | **S2** | **S3** | **S4** | **S5** |
| --- | --- | --- | --- | --- | --- | --- | --- | --- | --- | --- | --- | --- | --- | --- |
| 607.4362 | 662 | 7.0 | C_38_H_59_O_5_ | [M+H]^+^ | -0.8 | 20 | DG (C36:9) (C20:5/C16:4) | 607.4349 [M+H]^+^ ; 589.4270 [C_39_H_57_O_4_]^+^, 359.2584 [C_23_H_35_O_3_]^+^, 305.2110 [C_19_H_29_O_3_]^+^, 285.2212 [C_20_H_29_O]^+^ | 1.44E-13 |  |  |  |  |  |
| 903.5664 | 783 | 4.6 | C_55_H_75_N_4_O_7_ | [M+H]^+^ | -3.8 | 58.5 | Pheophytin derivative (Pheo +2ox) | 903.5681 [M+H]^+^ ; 885.5552 [C_55_H_73_N_4_O_6_]^+^; 625.2668 [C_35_H_37_N_4_O_7_]^+^ ; 607.2532 [C_35_H_35_N_4_O_6_]^+^ ; 581.2797 [C_34_H_37_N_4_O_5_]^+^; 565.2464 [C_33_H_33_N_4_O_5_]^+^ ; 503.2434 [C_32_H_31_N_4_O_2_]^+^ | 7.30E-06 |  |  |  |  |  |
| 490.3743 | 519 | 3.0 | C_26_H_52_NO_7_ | [M+H]^+^ | -0.2 | 9.2 | *lyso*-DGCC (C16:0) | 490.3744 [M+H]^+^ ; 313.2748 [C_19_H_37_O_3_]^+^ ; 132.1019 [C_6_H_14_NO_2_]^+^ ; 104.1069 [C_5_H_14_NO]^+^ | 0.0017 |  |  |  |  |  |
| 536.4361 | 773 | 2.6 | C_40_H_56_ | [M**^.^**]+ | 2.9 | 16.9 | *β*-carotene | 536.4391 [M**^.^**]^+^ ; 457.0.3757 [C_34_H_49_]^+^ ; 444,3754 [C_33_H_47_]^+^ ; 429.3513 [C_32_H_45_]^+^ ; 307.2419 [C_23_H_31_]^+^ ; 267.2109 [C_20_H_27_]^+^; 241.1950 [C_18_H_25_]^+^ ; 177.646 [C_13_H_21_]^+^; 133.1018 [C_10_H_13_]^+^ | 2.64E-07 |  |  |  |  |  |
| 659.4318 | 620 | 2.5 | C_42_H_59_O_6_ | [M+H]^+^ | -1.8 | 35.1 | Fucoxanthin ^d^ | 659.4292 [M+H]^+^ ; 411.1689 [C_20_H_35_O]^+^ ; 355.2436 [C_27_H_31_]^+^ ; 329.2255 [C_25_H_29_]^+^ ; 263.1789 [C_20_H_23_]^+^ ; 251.1799 [C_19_H_23_]^+^ , 213.1277 [C_15_H_17_O]^+^ ; 109.1012 [C_8_H_13_]^+^ | 9.30E-11 |  |  |  |  |  |
| 482.3476 | 596 | 2.2 | C_27_H_48_NO_6_ | [M+H]^+^ | -0.1 | 38.4 | GGG derivative | 482.3476 [M+H]^+^ ; 271.2430 [C_20_H_31_]^+^ ; 201.1638 [C_15_H_21_]^+^ ; 175.1476 [C_13_H_19_]^+^ ; 161.1323 [C_12_H_17_]^+^ ; 135.1172 [C_10_H_15_]^+^ ; 109.1007 [C_8_H_13_]^+^ | > 0.05 |  |  |  |  |  |
| 563.4675 | 685 | 2.1 | C_35_H_63_O_5_ | [M+H]^+^ | -0.9 | 44.1 | C_35_H_63_O_5_ | 563.4604 [M+H]^+^ ; [C_31_H_55_O_5_]^+^ ; 293.1748 [C_17_H_25_O_4_]^+^ ; 237.1122 [C_13_H_17_O_4_]^+^ ; 209.1173 [C_12_H_17_O_3_]+ | 4.20E-06 |  |  |  |  |  |
|  |  |  |  |  |  |  |  |  |  |  |  |  |  |  |
|  |  |  |  |  |  |  |  |  | Color code | | | | | |
|  |  |  |  |  |  |  |  |  | -2 | -1 | 0 | 1 | 2 | 3 |

^a^ Constructor statistical match factor (comparison of theoretical and experimental isotopic patterns); ^b^ Abbreviations: DG: diacylglycerol, DGCC : monoacylglyceryl-3-*O*-carboxy-(hydroxymethyl)-choline, GGG: geranylgeranylglycerol. ^d^ This identification was confirmed with a purified standard.

# Supplementary Figures

## **Figure S1.** Discrimination of heterotrophic prokaryotes by flow cytometry.

A successive 3-steps workflow was used. A: Samples were first screened for the presence of potential doublets or aggregates. Sample dilution was eventually adjusted in order to keep doublets below 5% of the total signal. B: Particles showing a red fluorescence (FL3) were excluded in order to keep only strict heterotrophs (*i.e.* presenting only the SYBR green-induced fluorescence). C: High side scatter signal harboring particles were excluded in order to enumerate only prokaryotes.


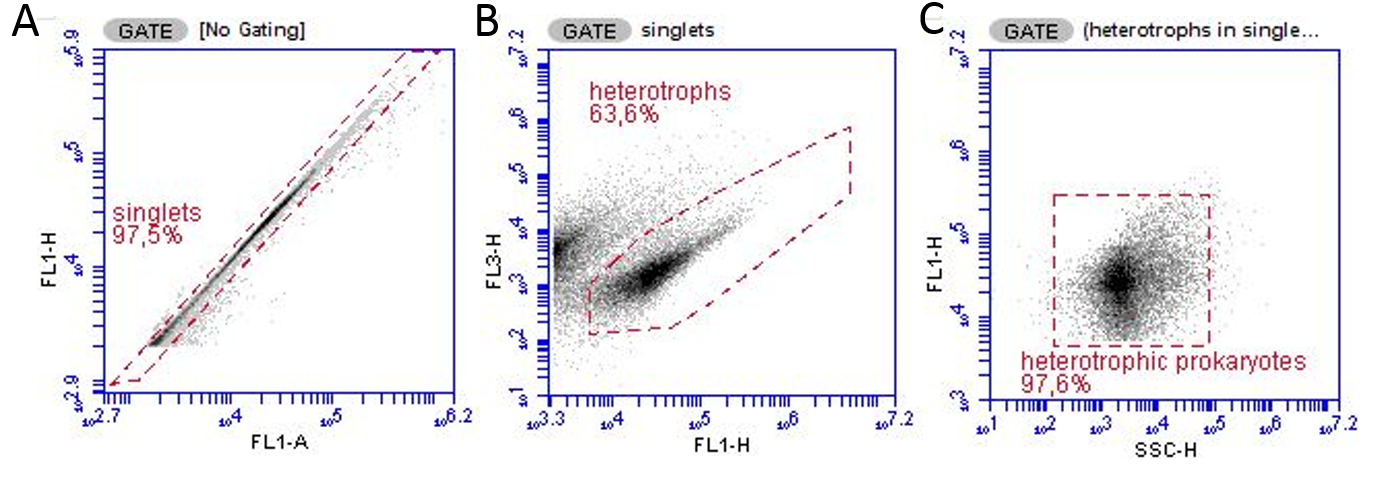


## Figure S2. Variations of thalli length (cm) conducted with all algal samples collected during the study and pictures of thalli collected at S1 (one replicate per month).


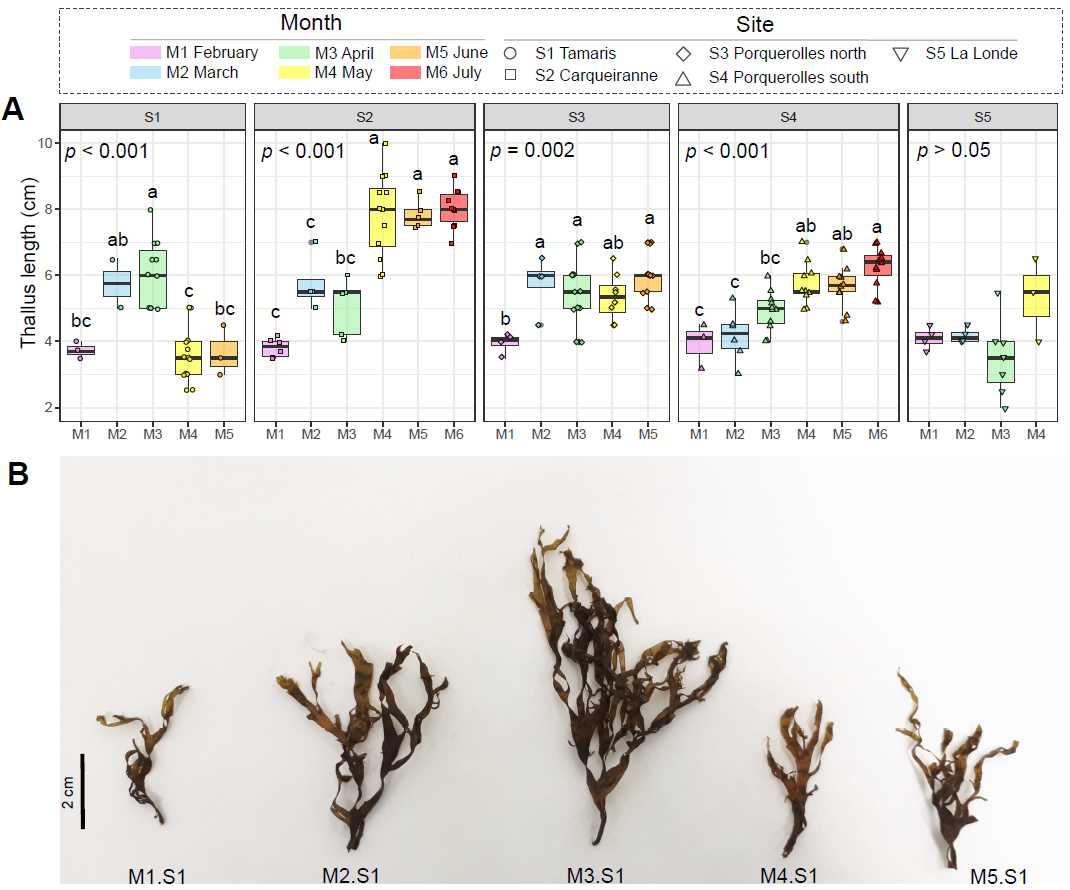


## Figure S3. Variations of cells densities at the surface of *T. atomaria* for each site (A) and heteroprokaryotic cell abundances in seawater samples (B). *p* values corresponded to results of one-way ANOVA using “Month” as the factor.


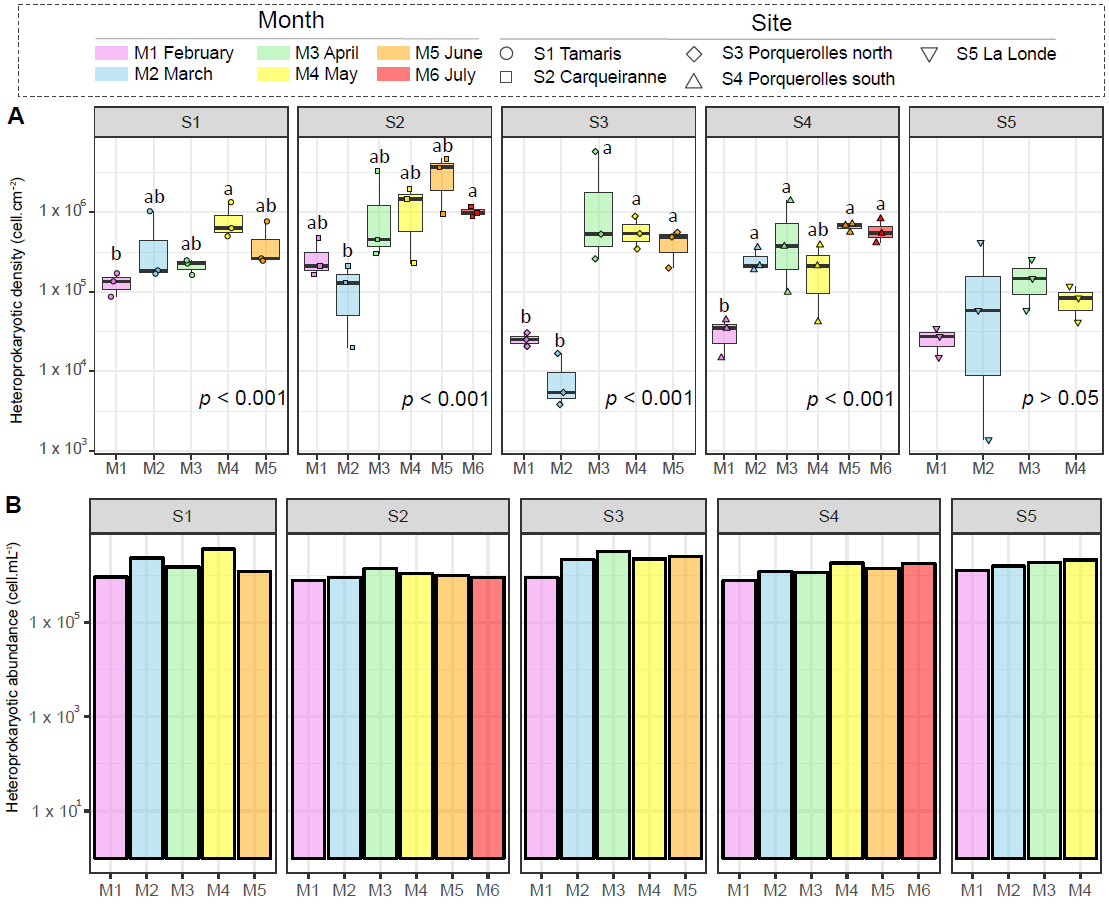


## Figure S4. Spatiotemporal dynamics of prokaryotic cell density (A), *α*-diversity (B), percentage of algal-core (C) and algal-enriched (D) taxa at the surface of *T. atomaria*. *p* values corresponded to results of one-way ANOVA using “Month” as factor.


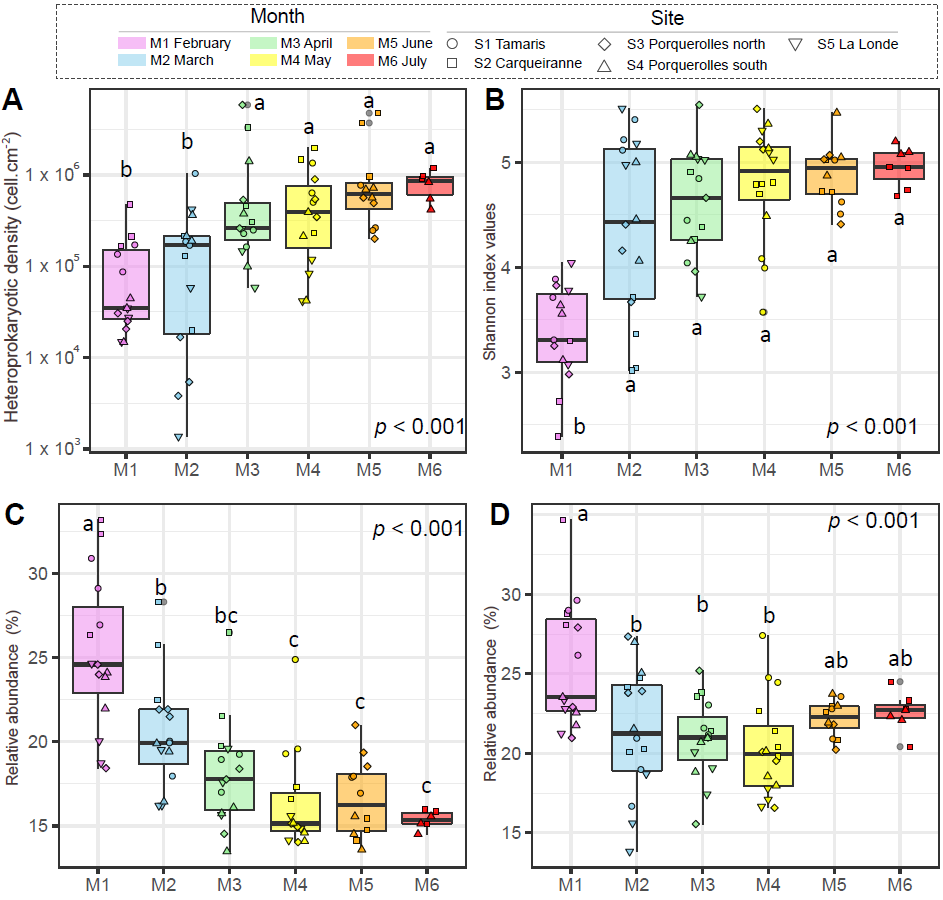


## Figure S5. NMDS (Bray-Curtis index) showing prokaryotic *β*-diversity among seawater, rocky and algal samples. *p* value corresponded to the result of a one-way ANOVA with seawater, rocky and algal samples as factors.


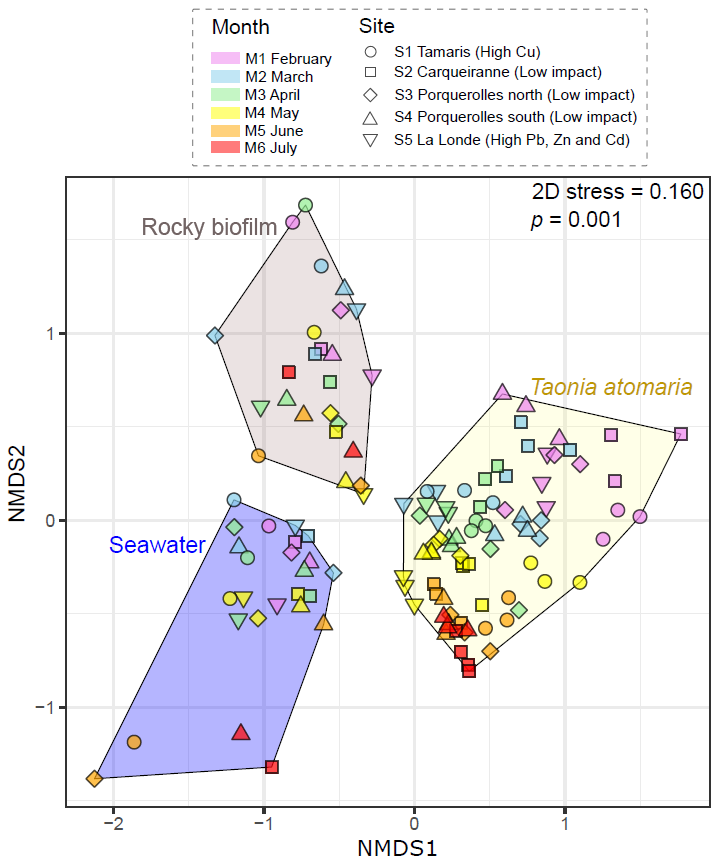


## Figure S6. NMDS (Bray-Curtis index) showing prokaryotic *β*-diversity of rocky biofilm (A) and seawater (B) samples. *p* values corresponded to results of one-way PERMANOVA using whether “Month” or “Site” as factors.


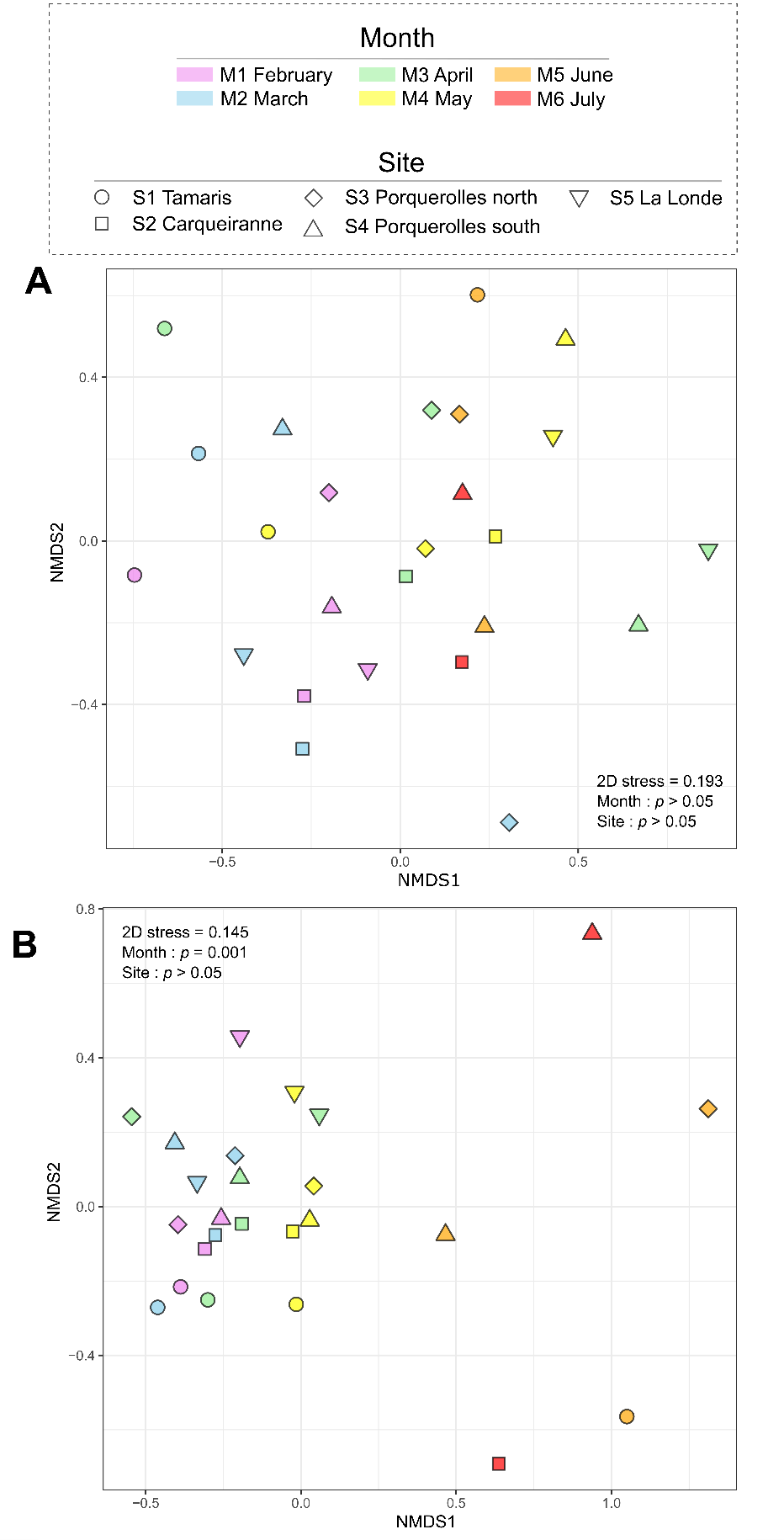


## Figure S7. Cladogram obtained from the LEfSe analysis built with the 16S rRNA gene dataset of all samples and revealing discriminant prokaryotic taxa specific to *T. atomaria*, to rocky biofilms and to seawater samples (LDA threshold set to 4).


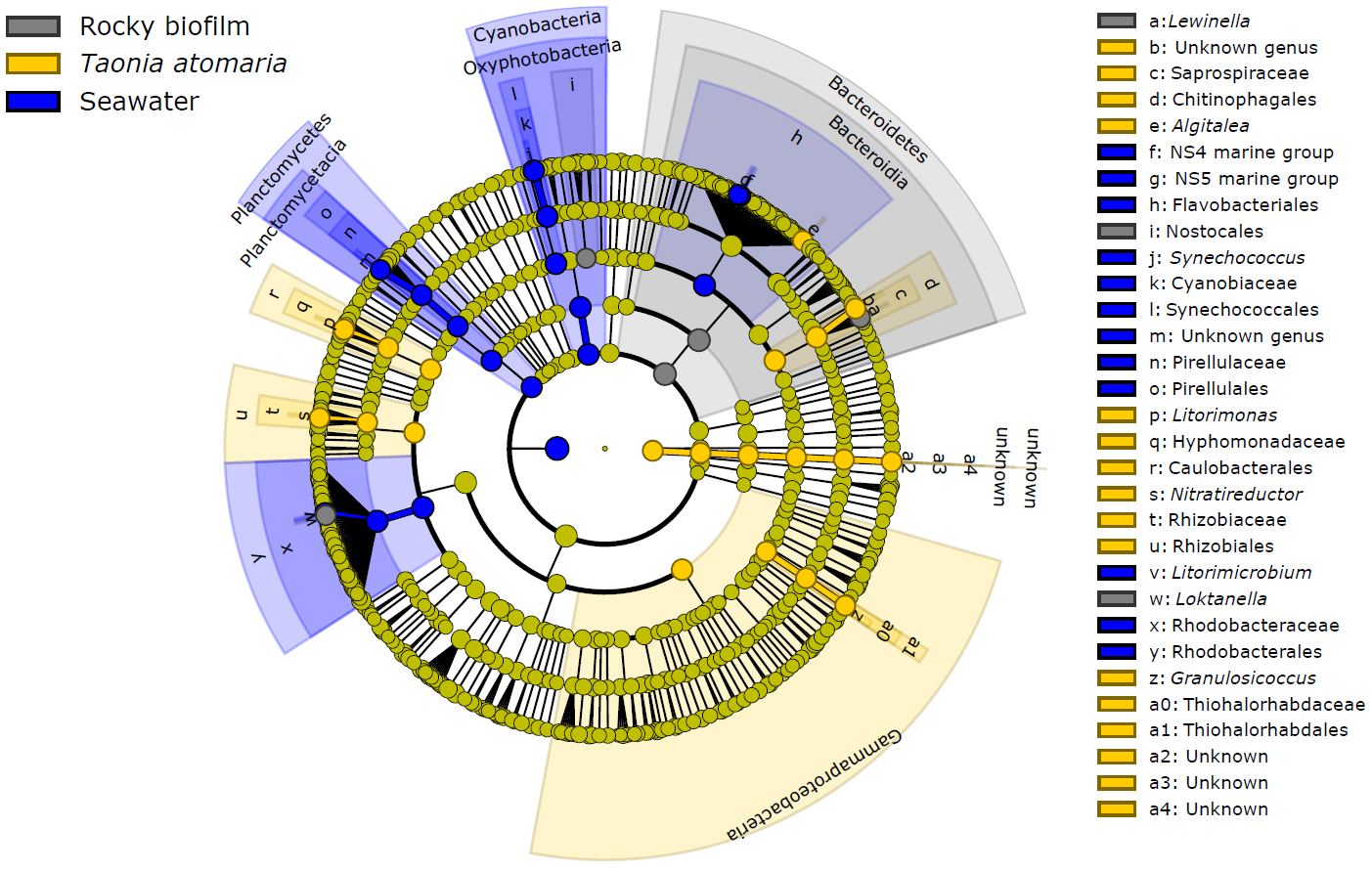


## Figure S8. Cladogram obtained from the LEfSe analysis built with the 16S rRNA gene dataset of *T. atomaria* samples and revealing discriminant epibacterial taxa specific to each sampling month (LDA threshold set to 4.5).


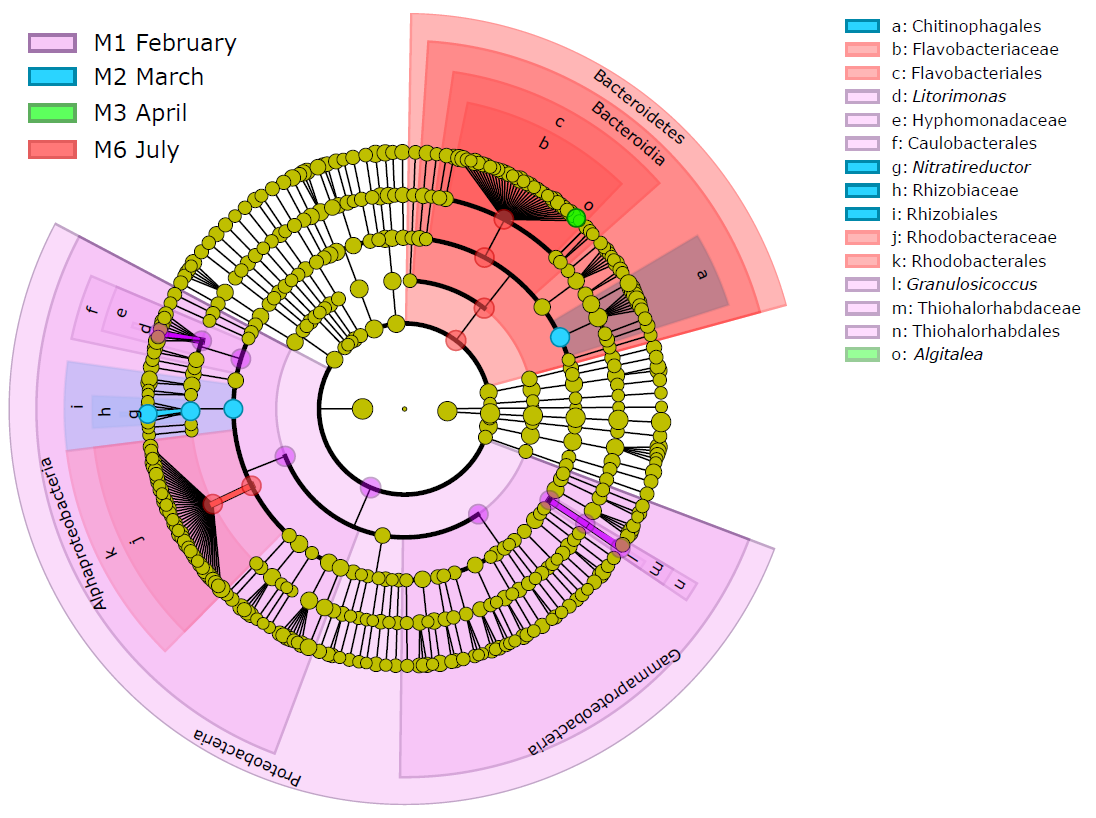


## Figure S9. Cladogram obtained from the LEfSe analysis built with the 16S rRNA gene dataset of *T. atomaria* samples and revealing discriminant epibacterial taxa specific to each sampling site (LDA threshold set to 3.5).


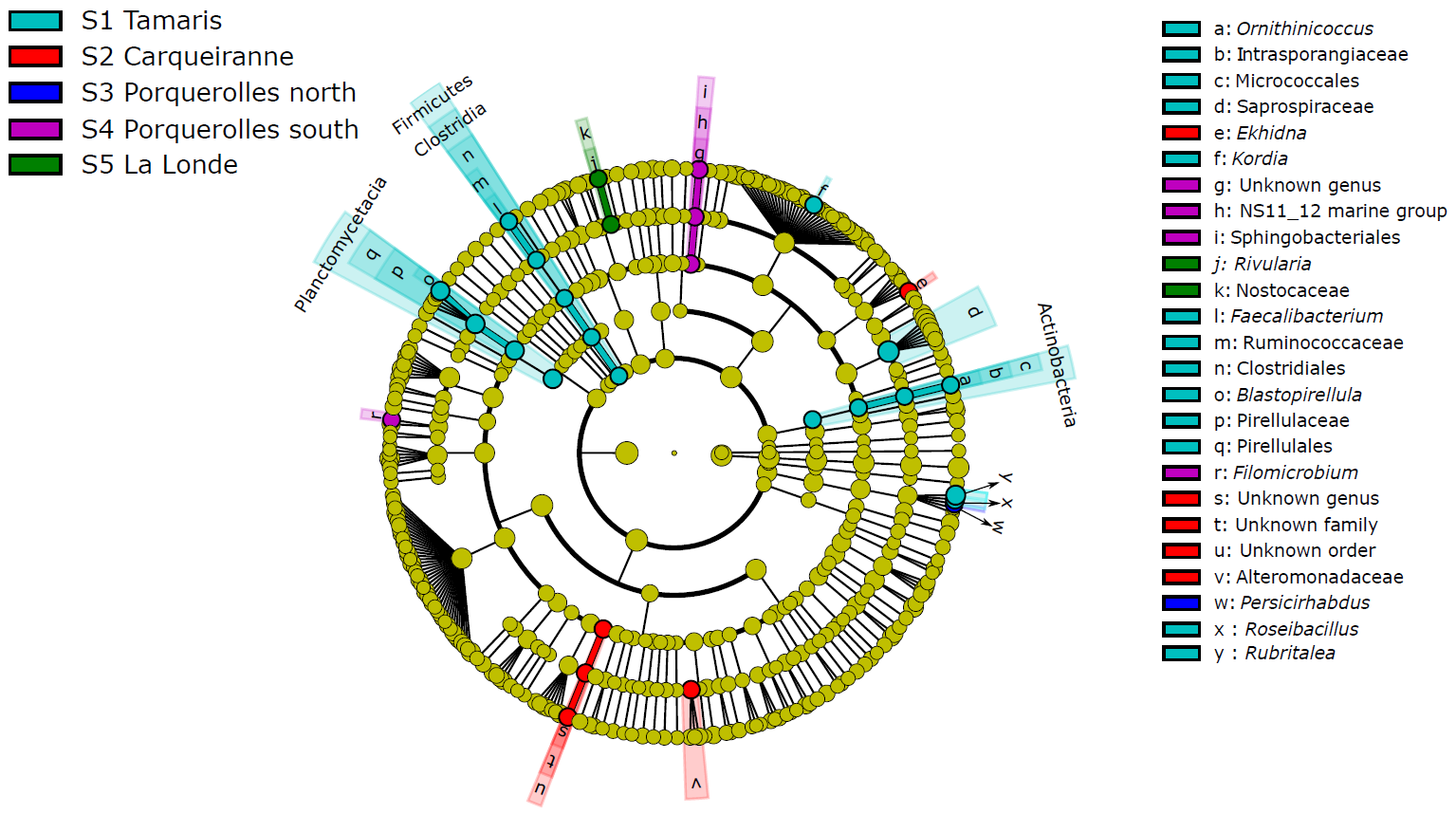


## Figure S10. Percentage of core community sequences (A) and algal-enriched community sequences (B). *: “Other” corresponded to unaffiliated families and families below 1%.

**A**


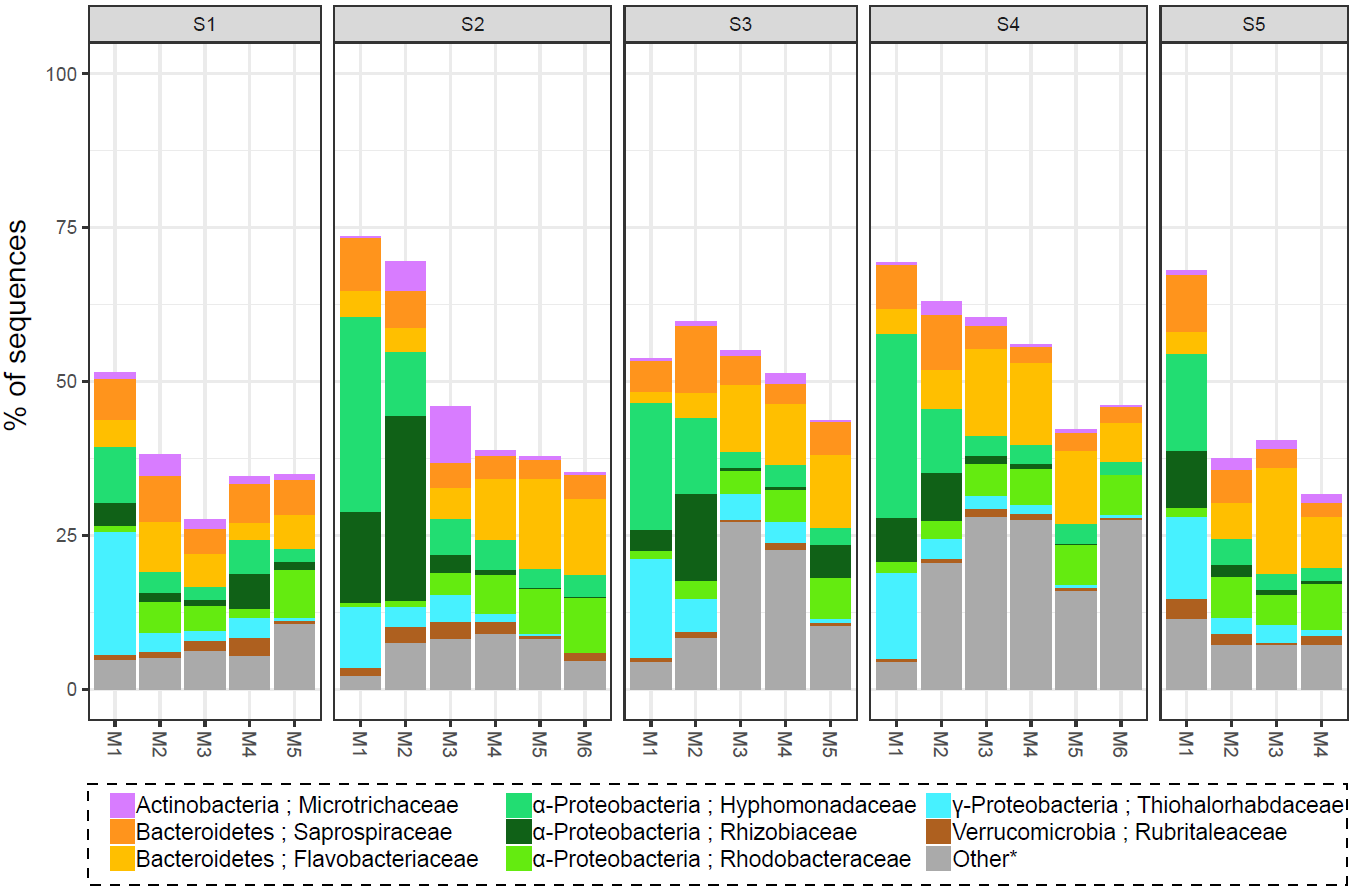


**B**


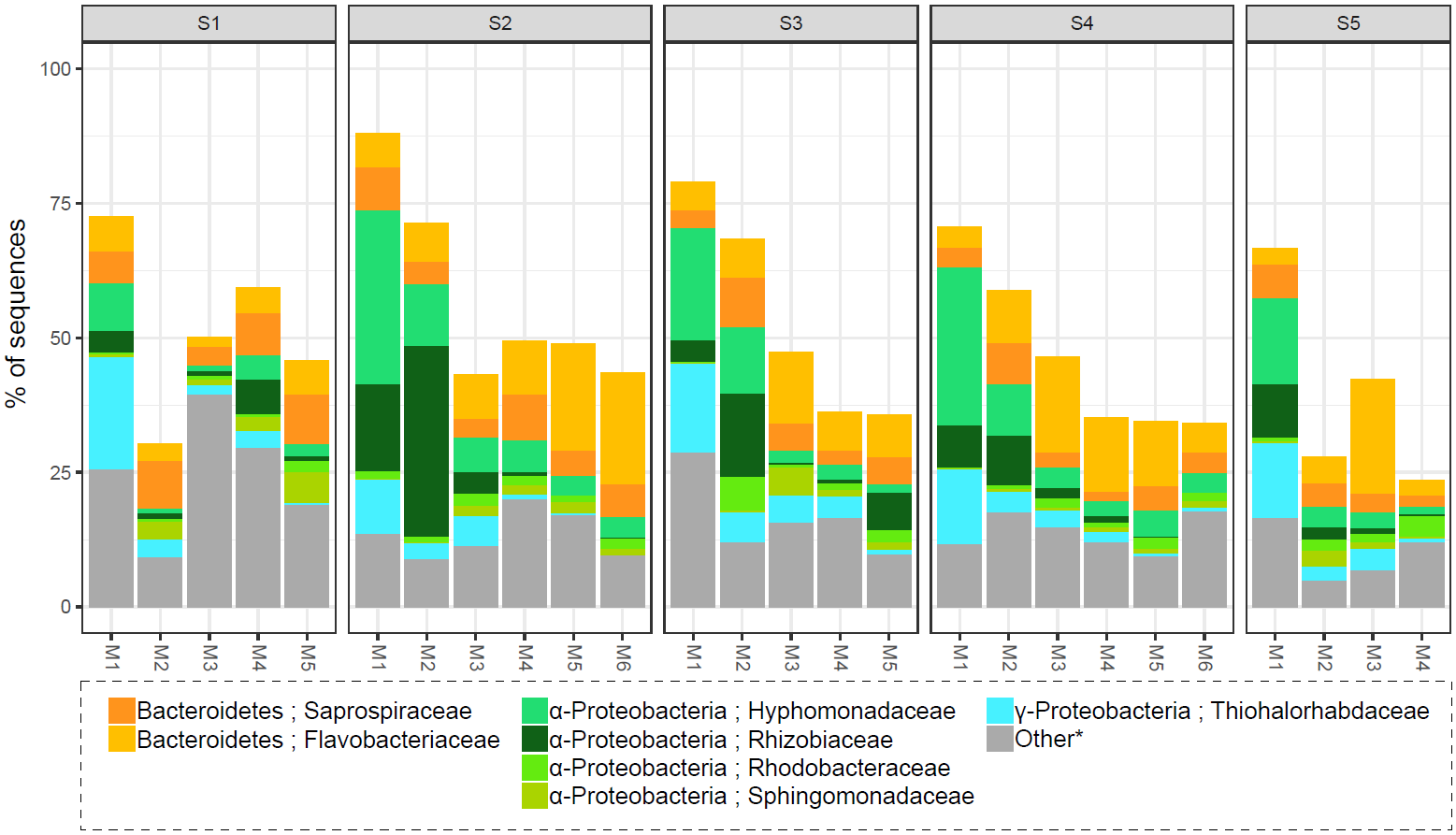


## Figure S11. Plots from the db-RDA scoring (A) environmental parameters together with algal samples (identical to Fig. 3B), and (B) environmental parameters together with OTUs represented by dashed circles. Dashed circles size was proportional to their relative percentage within the whole algal dataset. Dashed circles filled in pink corresponded to core OTUs, while those filled in dark blue were not affiliated as core members. The name of the 5 main core OTUs were annotated above their corresponding dashed circles.


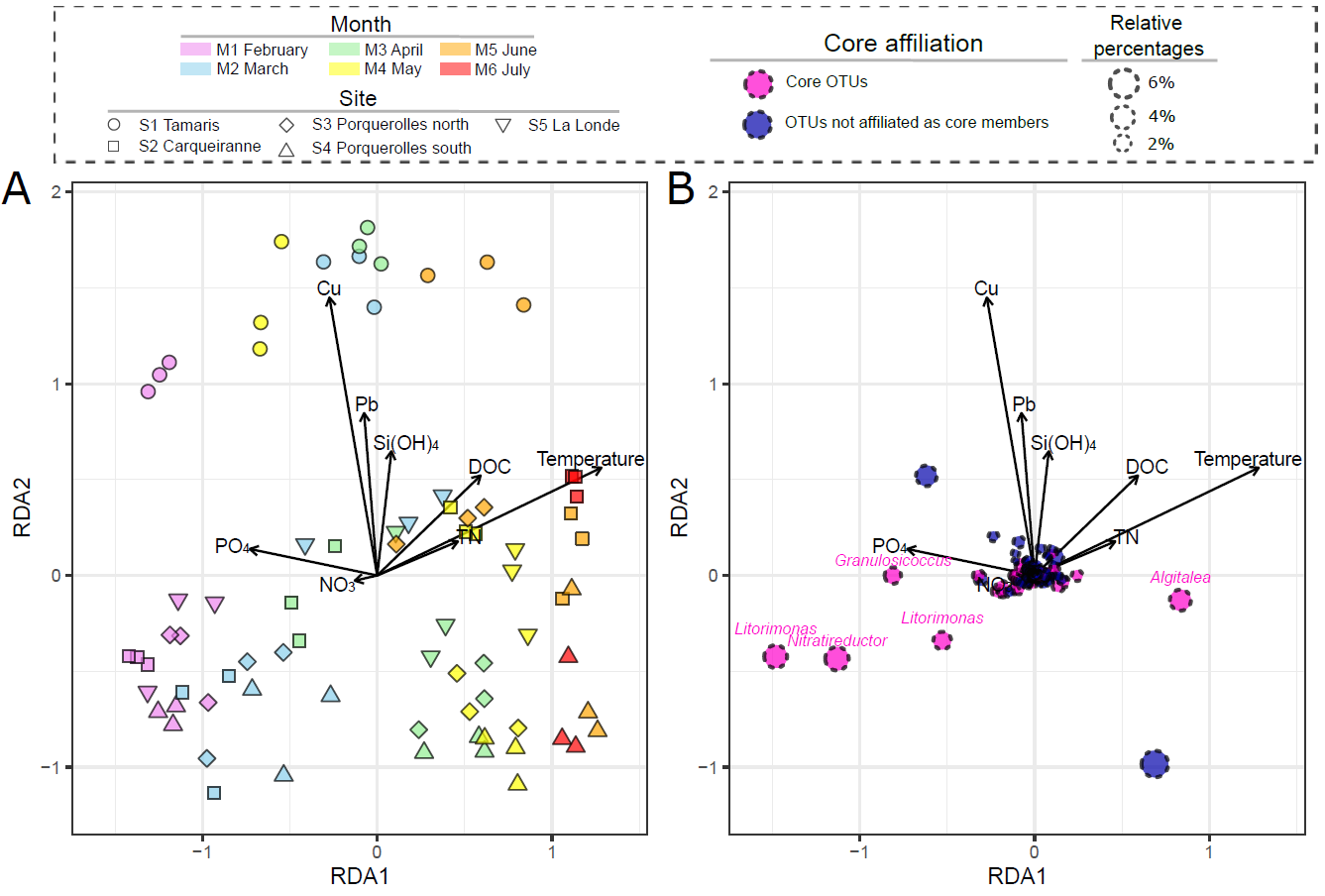


## Figure S12. GNPS molecular network built with LC-ESI-(+)-MS/MS data. Levels of annotations were attributed according to Schymanski et al., 2014. Abbreviations: DGTA: diacylglycerylhydroxymethyl-*N*,*N*,*N*-trimethyl-*β*-alanine, DGCC: monoacylglyceryl-3-O-carboxy-(hydroxymethyl)-choline, DG: diacylglycerol, MG: monoacylglycerol, GGG: geranylgeranylglycerol, DGDG : digalactosyldiacylglycerol, MGDG : monogalactosyldiacylglycerol

**
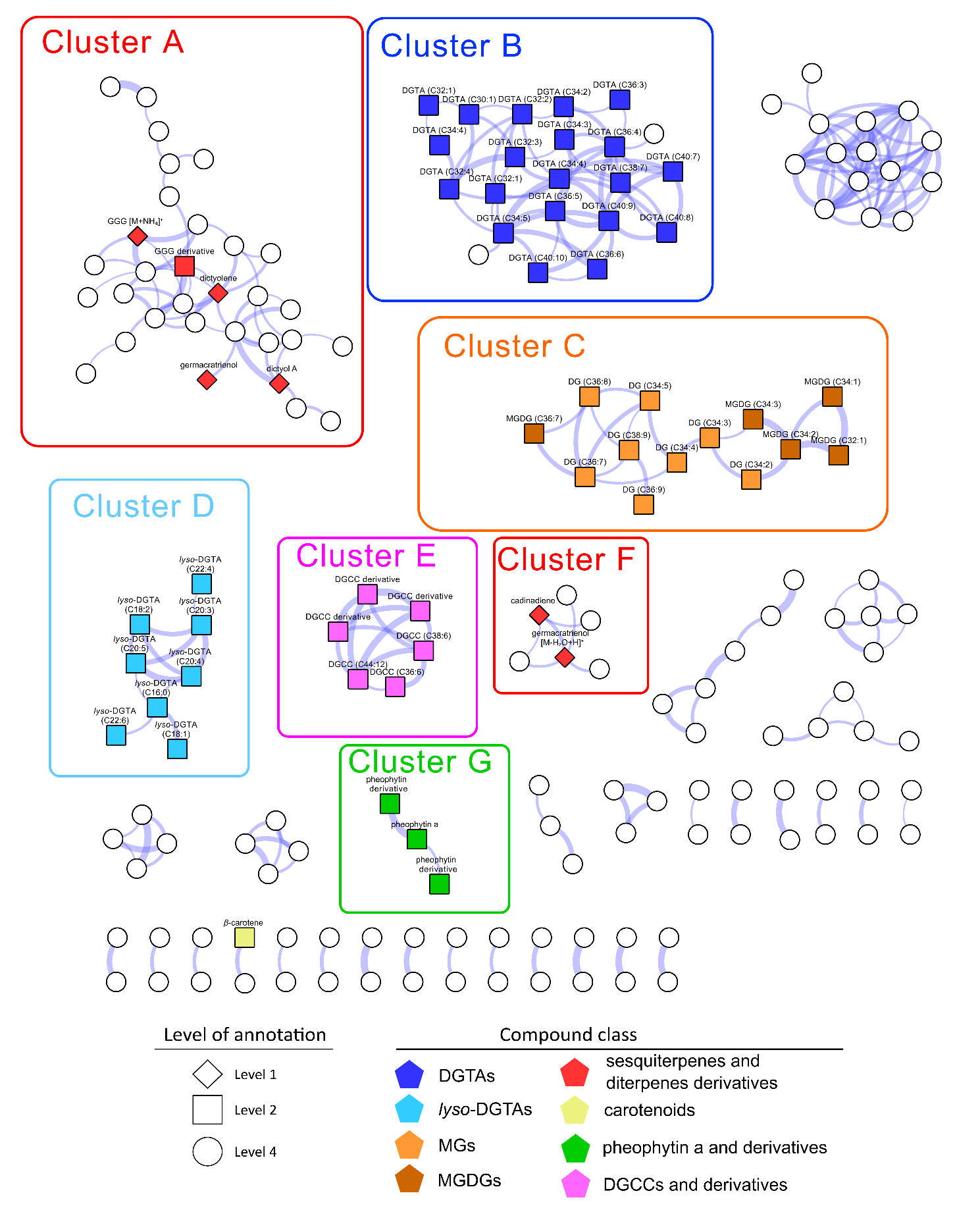
**

## Figure S13. Scatter plots showing the normalized concentration of DMSP, and the relative abundances of OTUs from the correlation network affiliated to Rhodobacteraceae (Figure S5), as a function of the temperature. Scatter plots were overlayed with smoothed conditional means plots using *geom_smooth()* function (loess regression, span = 0.6).


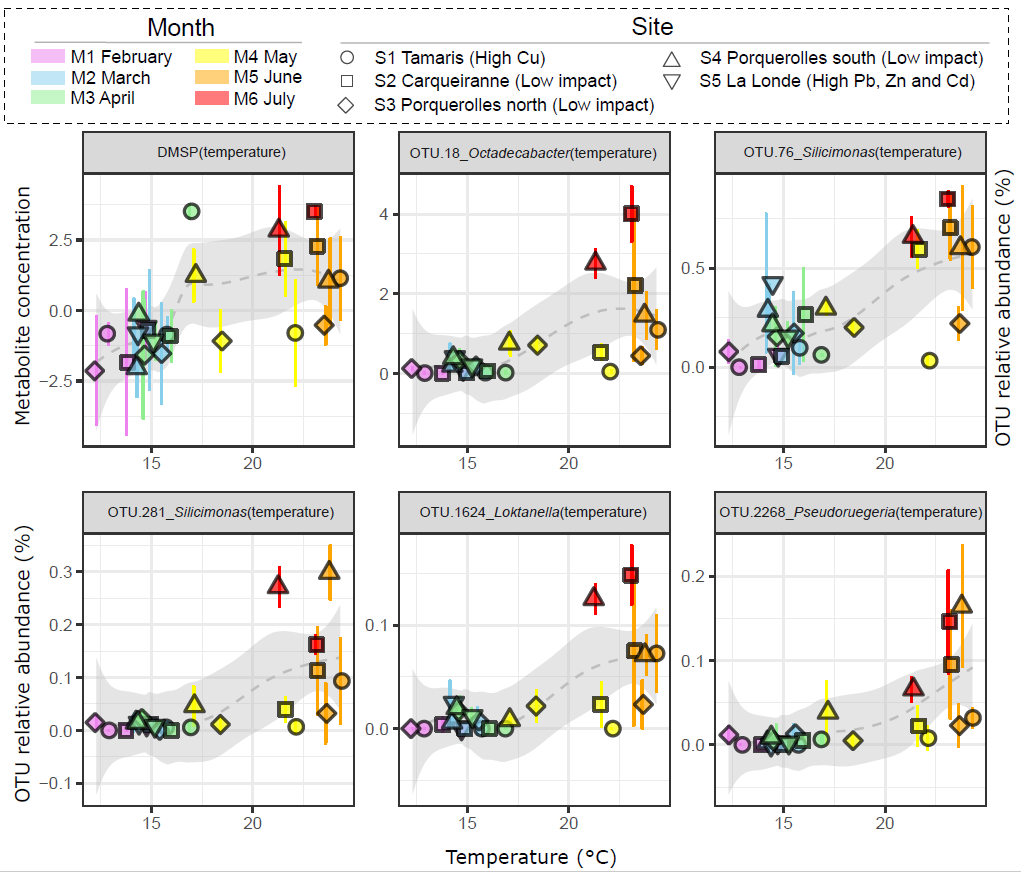


## Figure S14. Scatter plots showing the normalized concentration of fucoxanthin and β-carotene, and the relative abundances of OTUs from the correlation network affiliated to *Kordia* and *Roseibacillus* (Figure S5), as a function of the dissolved copper concentration. Scatter plots were overlayed with smoothed conditional means plots using *geom_smooth()* function (loess regression, span = 0.6).


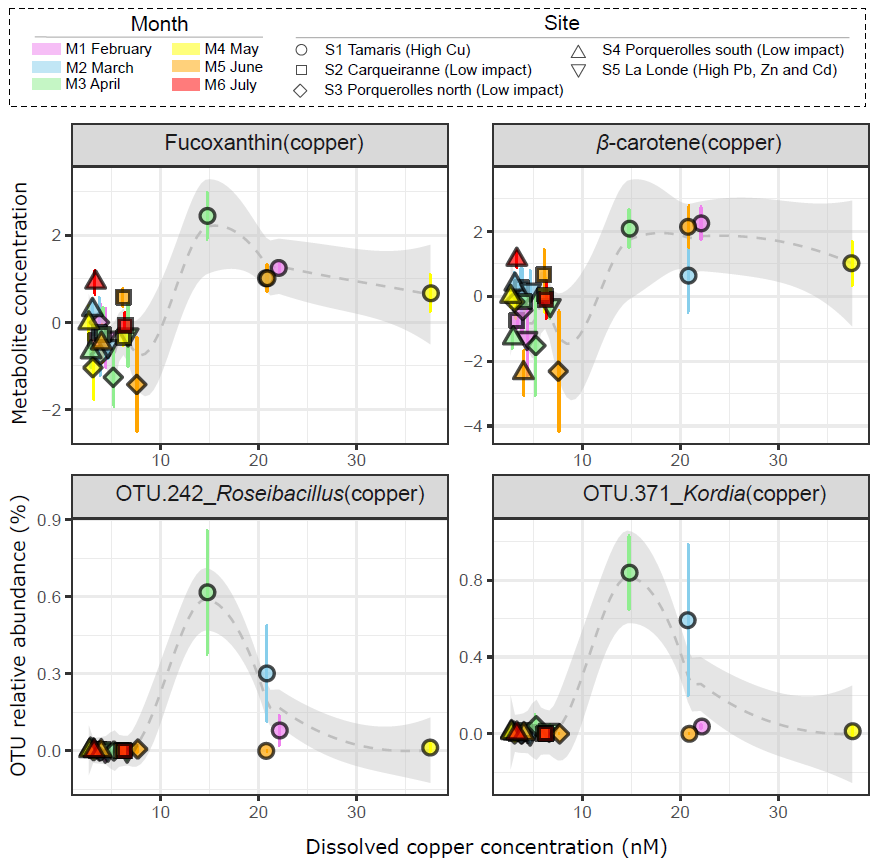


1. New affiliation: Institut Méditerranéen de Biodiversité et d'Ecologie marine et continentale (IMBE), UMR CNRS-IRD-Avignon Université-Aix-Marseille Université, Avignon, France. [↑](#footnote-ref-1)
